# Efficacy and safety of iguratimod in the treatment of rheumatic and autoimmune diseases: a meta-analysis and systematic review of 84 randomized controlled trials

**DOI:** 10.3389/fphar.2023.1189142

**Published:** 2023-12-07

**Authors:** Liuting Zeng, Qi He, Ying Deng, Yuwei Li, Junpeng Chen, Kailin Yang, Yanfang Luo, Anqi Ge, Xiaofei Zhu, Zhiyong Long, Lingyun Sun

**Affiliations:** ^1^ Department of Rheumatology and Immunology, Nanjing Drum Tower Hospital, Chinese Academy of Medical Sciences and Peking Union Medical College, Graduate School of Peking Union Medical College, Nanjing, China; ^2^ People’s Hospital of Ningxiang City, Ningxiang, China; ^3^ Hunan University of Science and Technology, Xiangtan, China; ^4^ Key Laboratory of Hunan Province for Integrated Traditional Chinese and Western Medicine on Prevention and Treatment of Cardio-Cerebral Diseases, School of Integrated Chinese and Western Medicine, Hunan University of Chinese Medicine, Changsha, China; ^5^ Department of Nephrology, The Central Hospital of Shaoyang, Shaoyang, China; ^6^ The First Hospital of Hunan University of Chinese Medicine, Changsha, China; ^7^ Fudan University, Shanghai, China; ^8^ Department of Rehabilitation Medicine, Guangzhou Panyu Central Hospital, Guangzhou, China; ^9^ Department of Rheumatology and Immunology, The First Affiliated Hospital of Anhui Medical University, Anhui, China

**Keywords:** autoimmune disease, iguratimod, rheumatoid arthritis, ankylosing spondylitis, primary Sjögren’s syndrome, autoimmune disease with interstitial pneumonia, systematic review, meta-analysis

## Abstract

**Objective:** To evaluate efficacy and safety of iguratimod (IGU) in the treatment of rheumatic and autoimmune diseases.

**Methods:** Databases such as Pubmed, Embase, Sinomed were searched (as of July 2022) to collect randomized controlled trials (RCTs) of IGU in the treatment of rheumatic and autoimmune diseases. Two researchers independently screened the literature, extracted data, assessed the risk of bias of the included literature, and performed meta-analysis using RevMan 5.4 software.

**Results:** A total of 84 RCTs and 4 types of rheumatic and autoimmune diseases [rheumatoid arthritis (RA), ankylosing spondylitis (AS), primary Sjögren’s syndrome (PSS) and Autoimmune disease with interstitial pneumonia]. Forty-three RCTs reported RA and showed that IGU + MTX therapy can improve ACR20 (RR 1.45 [1.14, 1.84], *p* = 0.003), ACR50 (RR 1.80 [1.43, 2.26], *p* < 0.0000), ACR70 (RR 1.84 [1.27, 2.67], *p* = 0.001), DAS28 (WMD −1.11 [−1.69, −0.52], *p* = 0.0002), reduce ESR (WMD −11.05 [−14.58, −7.51], *p* < 0.00001), CRP (SMD −1.52 [−2.02, −1.02], *p* < 0.00001), RF (SMD −1.65 [−2.48, −0.82], *p* < 0.0001), and have a lower incidence of adverse events (RR 0.84 [0.78, 0.91], *p* < 0.00001) than the control group. Nine RCTs reported AS and showed that IGU can decrease the BASDAI score (SMD −1.62 [−2.20, −1.05], *p* < 0.00001), BASFI score (WMD −1.07 [−1.39, −0.75], *p* < 0.00001), VAS (WMD −2.01 [−2.83, −1.19], *p* < 0.00001), inflammation levels (decreasing ESR, CRP and TNF-α). Thirty-two RCTs reported PSS and showed that IGU can reduce the ESSPRI score (IGU + other therapy group: WMD −1.71 [−2.44, −0.98], *p* < 0.00001; IGU only group: WMD −2.10 [−2.40, −1.81], *p* < 0.00001) and ESSDAI score (IGU + other therapy group: WMD −1.62 [−2.30, −0.94], *p* < 0.00001; IGU only group: WMD −1.51 [−1.65, −1.37], *p* < 0.00001), inhibit the inflammation factors (reduce ESR, CRP and RF) and increase Schirmer’s test score (IGU + other therapy group: WMD 2.18 [1.76, 2.59], *p* < 0.00001; IGU only group: WMD 1.55 [0.35, 2.75], *p* = 0.01); The incidence of adverse events in IGU group was also lower than that in control group (IGU only group: RR 0.66 [0.48, 0.98], *p* = 0.01). Three RCTs reported Autoimmune disease with interstitial pneumonia and showed that IGU may improve lung function.

**Conclusion:** Based on current evidence, IGU may be a safe and effective therapy for RA, AS, PSS and autoimmune diseases with interstitial pneumonia.

**Systematic Review Registration**: (CRD42021289489).

## 1 Introduction

The pathogenesis of rheumatic immune diseases is complex, and it is an inflammatory disease that may lead to impaired immune system due to various reasons (involving the musculoskeletal system, joints and their surrounding soft tissues, *etc.*) (Konig, 2020; Adelowo et al., 2021). In recent years, the prevalence of rheumatic immune diseases has been on the rise (Hyrich and Machado, 2021), among which rheumatoid arthritis (RA), systemic lupus erythematosus (SLE) and ankylosing spondylitis (AS) are more common and have certain disability (Charoenngam, 2021) ]. Meanwhile, with the progression of the disease, most patients may develop complications such as kidney, iris, skin, heart and other organ damage (van der Woude and van der Helm-van Mil, 2018; Dai et al., 2021). Especially in active disease, there may be radioactive progression, and severe cases may lead to joint deformity and even loss of self-care function in life (Otón and Carmona, 2019). Therefore, rheumatic immune diseases with high disease activity will generate a great economic burden for both society and patients (Otón and Carmona, 2019). The current treatments for rheumatic diseases and autoimmune diseases are precision medicine based on drugs (Aletaha, 2020; Radu and Bungau, 2021), with the aim of controlling the progression of inflammation and reducing inflammatory damage (Winthrop, 2017; Aletaha and Smolen, 2018). It mainly includes traditional synthetic DMARDs, biologics DMARDs and synthetic targeted DMARDs (Goodman, 2015). Among them, biological DMARDs can be divided into two categories: biological agents (bDMARDs) and synthetic targeted (tsDMARDs) (Akram et al., 2021). bDMARDs include the tumor necrosis factor inhibitor class of adalimumab, infliximab, etanercept, and the IL-6 antagonist tocilizumab. tsDMARDs include the Janus kinase (JAK) inhibitor tofacitinib (Winthrop, 2017). Although the efficacy of the above drugs has been proven, their high prices make it impossible for patients in developing countries, including China, to benefit (Drosos et al., 2020). Studies have shown that patients in developed countries are also becoming increasingly prominent due to poor compliance and high recurrence rates related to medication problems (Tanaka, 2016; Ghabri et al., 2020). Traditional DMARDs are widely used in clinic because of their acceptable side effects and reasonable price. For example, methotrexate (MTX) is the most widely used DMARDs for the treatment of RA (Wang W. et al., 2018). Because of its effectiveness, acceptable side effects, and reasonable price, ACR recommends it as the first-choice drug in the initial treatment regimen for RA patients (Cronstein and Aune, 2020). However, there are still about 30%–40% of patients who are insensitive to MTX treatment, have poor treatment effect, or fail to benefit from it because of side effects (Cronstein and Aune, 2020). Strand et al. reported that the ACR50 of MTX in RA was 46%, and the ACR70 was 23% (Strand et al., 1999). According to multiple clinical trials, the combined use of DMARDs is one of the effective ways to improve the efficacy (Kremer et al., 2002; Ichikawa et al., 2005; Capell et al., 2007).

Iguratimod (IGU) is a new type of small molecule DMARDs developed in Japan. As an immunomodulator, through immunomodulation, it reduces immune response, inhibits collagenous arthritis, and relieves the destruction of bone and cartilage tissue (Li et al., 2013; Mizutani et al., 2021). IGU can also inhibit the activity of nuclear factors, thereby inhibiting the production of inflammatory cytokines, IL-1, IL-6, IL-8, and TNF, and inhibiting the production of immunoglobulins to exert anti-inflammatory, anti-immune, and anti-inflammatory effects. (Li et al., 2013; Xie S. et al., 2020). Several studies have shown that IGU has good efficacy in rheumatic diseases and autoimmune diseases, such as improving RA, AS, systemic lupus erythematosus, IG4-RD, pulmonary interstitial disease, primary Sjögren’s syndrome (PSS), *etc.* (Harjacek, 2021; Pu et al., 2021; Zeng et al., 2022a). In clinical practice, more and more rheumatologists use IGU to treat rheumatic and autoimmune diseases, but its efficacy and safety are still uncertain. Therefore, we collected randomized controlled trials (RCTs) of IGU in the treatment of rheumatic and autoimmune diseases in order to conduct a systematic review and meta-analysis of its efficacy and safety.

## 2 Materials and methods

### 2.1 Protocol

This systematic review and meta-analysis were conducted strictly in accordance with the protocol registered in PROSPERO (CRD42021289489) and PRISMA-guidelines (see Supplementary Materials) (Page et al., 2021).

### 2.2 Search criteria

#### 2.2.1 Study design

All RCTs on IGU for rheumatic and autoimmune diseases were included. There are no restrictions on publication year, publication language, publication journal, *etc.*

#### 2.2.2 Participants

Patients were diagnosed with any rheumatic and autoimmune diseases by accepted criteria.

#### 2.2.3 Intervention methods

The experimental group was treated with IGU, which was administered orally. The course of treatment and the dose were not limited, and it could be combined or not combined with other therapies. The control group is therapy that does not contain IGU, including but not limited to placebo, conventional therapy, *etc.*

#### 2.2.4 Outcomes

Outcomes are the disease activity indices (such as BASDAI and ACR20), inflammatory factor indicators (such as ESR, CRP, RF) and adverse events.

#### 2.2.5 Exclusion criteria

1) Duplicate publications; 2) Unable to obtain full text or incomplete data; 3) Reviews, case reports, animal experiments, *etc.*,; 4) Retracted studies; 5) observational studies.

### 2.3 Search strategy

Pubmed, Wanfang Database, Web of Science, China National Knowledge Infrastructure (CNKI), Sinomed, VIP Database, Medline Complete, Embase were searched for literature on IGU for the treatment of rheumatic and autoimmune diseases. The retrieval time is from inception to 1 July 2022. We also searched ClinicalTrials.gov and Cochrane Library. The search strategy was shown in Supplementary Table S1.

### 2.4 Data collection and analysis

#### 2.4.1 Literature screening and data extraction

Two researchers independently screened the title and abstract of the articles revealed from the search. Then, they screened the full text of the relevant articles based on search criteria. Finally, the two researchers reconciled the results and negotiated inconsistencies through discussions with all researchers (Deeks et al., 2020a). Then two researchers independently extracted the basic information, medication regimen, course of treatment, and outcome indicators of eligible RCTs. For inconsistencies, the solution is the same as before.

#### 2.4.2 Quality assessments

The risk of bias assessment of the included trials was independently performed by two investigators. The Cochrane Collaboration’s tool was used for assessing risk of bias (Deeks et al., 2020b). The content of the evaluation mainly includes: 1) Whether the method of random allocation is described; 2) Whether the allocation concealment is sufficient; 3) Whether the blind method is used; 4) Whether the withdrawal from the experiment and the loss to follow-up are completely described; 5) Whether the outcome indicators are selectively reported; 6) Whether there are other factors that may affect the quality of the trial. According to the Cochrane Handbook, the above items were judged as “Yes” (low risk of bias), “No” (high risk of bias), and “Unclear” (unclear risk of bias) (Deeks et al., 2020b).

### 2.5 Statistical analysis

Revman 5.4 software were utilized for meta-analysis (Deeks et al., 2020c). For dichotomous variables data, use the risk ratio (RR). For continuous variables data, when the results of different experiments are expressed in the same unit of measurement, the weighted mean difference (WMD) is used; when the results of the experiments are expressed in different units of measurement, the standard mean difference (SMD) is used. Effect sizes were expressed as 95% confidence intervals (CI). To analyze the heterogeneity between results, the chi-square test was employed. If heterogeneity was deemed small (*p* > 0.1, I2<50%), the fixed-effects model was utilized for analysis. Otherwise, the random-effects model was used. STATA 15 was used to detect publication bias with the Egger method (for continuous variables) and Harbord methods (for dichotomous variables) for outcomes with RCTs ≥4. *p* > 0.1 is considered indicative of no publication bias. The level of evidence of efficacy indicators (such as ACR and BASFI) and adverse events was evaluated by the GRADE tool (GRADEpro, 2015), following the GRADE handbook (Schünemann et al., 2013).

## 3 Results

### 3.1 Literature search results

**FIGURE 1:**
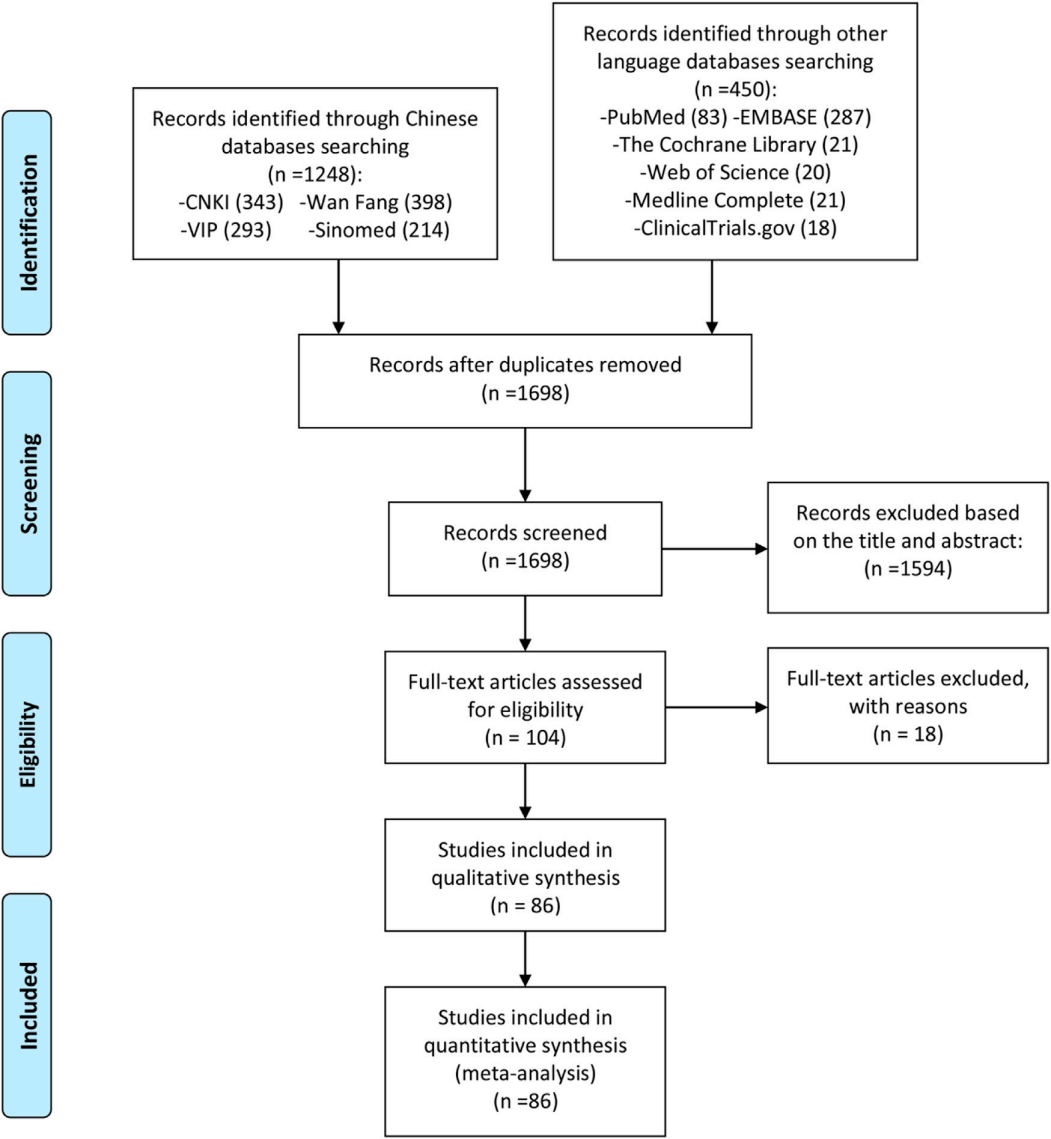
Flow diagram of clinical research.

A total of 1,698 preliminary related literature were detected in this study, and a total of 1,594 literature that did not conform to the research type and content were excluded. After the primary screening, 104 records were obtained. According to the inclusion and exclusion criteria and the completeness of the literature information, 18 records were excluded from the second screening after reading the full text (GuifengLi, 2014; He et al., 2015; Okamura et al., 2015; Meng et al., 2016a; Lin, 2016; Yoshioka et al., 2016; Zhu et al., 2016; Wang, 2017; Wang et al., 2017; Luo Y. et al., 2018; Wang X. et al., 2018; Huang and Ma, 2018; Luo et al., 2019; Shang et al., 2019; Suto et al., 2019; Gu et al., 2020; ManXie, 2020; Xu et al., 2021), and 86 records [(GuifengLi, 2014; Lü et al., 2008; Tian and Tao, 2017; Qi et al., 2019; Hara et al., 2014; Ishiguro et al., 2013; Li L. et al., 2019; Hu, 2014; Xia et al., 2020; Xia et al., 2016; Lu, 2014; Zhao et al., 2016; Shi et al., 2015; Meng et al., 2015; Bi, 2019; Zhang, 2018; Li et al., 2016; Mo et al., 2018; Duan et al., 2015; Xiong and GengGuanghui, 2020; Shang, 2014; Mo and Ma, 2015; Tian et al., 2020; Xu B. et al., 2015; Xu LM. et al., 2017; Yan and Wang, 2018; Fan et al., 2020; Meng et al., 2016b; Wang L. et al., 2019; Meng et al., 2017; Ju et al., 2020; Zhao and Hao, 2018; Li and WH, 2020; Xu YM. et al., 2015; Chen et al., 2018; Zhao et al., 2017a; Deng, 2017; Xie et al., 2018; Rao et al., 2014; Wang et al., 2022; Dai et al., 2022; Sun and Li, 2022; Wu et al., 2022; Dong Zhang et al., 2019; Qiu et al., 2016; Yuan et al., 2020; Pang et al., 2020; Lin et al., 2019; Xu et al., 2019; Zeng et al., 2016; Li Y. et al., 2021; Bai et al., 2021; Li X. et al., 2021; Gu, 2020; Jiang et al., 2014; Zhao, 2019; Lu and Zhang, 2021; Li et al., 2020; Zhang, 2019; Jia, 2020; Yu, 2020; Shao et al., 2020; Chen et al., 2022; Donghui, 2019; Zhang and Shen, 2019; Jiang et al., 2016; Xie H. et al., 2020; Jiang et al., 2020; Bai and Jiao, 2019; Rao et al., 2022; Ding et al., 2022; Xu D. et al., 2017; Zhang et al., 2019; Luo Q. et al., 2018; Wang Y. et al., 2019; Zhao, 2020; Liang et al., 2021; Li et al., 2018; Jiang, 2021; Yi, 2018; Zhuang, 2020; Xia et al., 2017; Gu, 2022; Liu, 2022; Zhuang et al., 2021; Du et al., 2008; Lu et al., 2009) were finally included in the quantitative and qualitative analysis of the review. The literature screening process and results are shown in Figure 1.

### 3.2 Description of included trials

Two records (Ishiguro et al., 2013; Hara et al., 2014) came from the same RCT and were therefore recorded as Hara et al., 2014 (Ishiguro et al., 2013; Hara et al., 2014). Two records (Lu, 2014; Xia et al., 2016) came from the same RCT and were therefore recorded as Lu (2014); Xia et al. (2016). Therefore, 86 records actually involve 84 RCTs. In some RCTs, there were 2 experimental groups, and to match them, the control group was split into 2 equal parts with half the population each, and labeled as groups a and b (e.g., Xu et al., 2015a and Xu YM. et al., 2015). The included RCTs involved 4 rheumatic and autoimmune diseases (RA, AS, PSS and Autoimmune disease with interstitial pneumonia). The details of study characteristics are presented in Table 1.

**TABLE 1:** The characteristics of the included studies.

Disease	Study	Sample size		Intervention		Relevant outcomes	Mean age (years)		Duration
		Trial group	Control group	Trial group	Control group		Trial group	Control group	
RA	Lü et al. (2008)	185	95	a: IGU 25 mg Qd; b: 25 mg Bid	Placebo	American college of rheumatology (ACR)20, ACR50, ACR70, Erythrocyte sedimentation rate (ESR), C-reactive protein (CRP), rheumatoid factor (RF), adverse events	a: 48.05 ± 10.30; b: 46.98 ± 10.93	47.46 ± 10.30	24 weeks
	Tian and Tao (2017)	58	58	IGU 25 mg Bid + MTX 10 mg once or twice a week	MTX 10 mg once or twice a week	Disease activity score (DAS)28, ESR, CRP, adverse events	52.6 ± 7.6	49.7 ± 8.4	24 weeks
	Qi et al. (2019)	40	40	IGU 25 mg Bid + MTX 7.5 mg once a week at the beginning, gradually increase to 10 mg within 4 weeks	MTX 7.5 mg once a week at the beginning, Gradually increase to 10 mg within 4 weeks	ACR20, ACR50, ACR70, ESR, CRP, adverse events	25–65		24 weeks
	Ishiguro et al. (2013), Hara et al. (2014)	164	68	IGU 25 mg Qd for the first 4 weeks of the extension period 25 mg Bid for the subsequent 20 weeks + MTX 6–8 mg once a week	MTX 6–8 mg once a week + placebo	ACR20, ACR50, ACR70, CRP, RF, DAS28, adverse events	54.8 ± 9.9	53.5 ± 10.0	24 weeks
	Li et al. (2019a)	51	51	IGU 25 mg Bid + MTX 15 mg once a week	MTX 15 mg once a week	Adverse events	74.16 ± 2.42	74.32 ± 2.52	15 weeks
	Hu (2014)	20	20	IGU 25 mg Bid	MTX 10 mg once a week	DAS28, ACR20, adverse events	47.3 ± 13.5	46.2 ± 15.8	24 weeks
	Du et al. (2008)	326	163	a: IGU 25 mg for the first 4 weeks and 50 mg for the subsequent 20 weeks; b: IGU 25 mg Bid	MTX 10 mg/week for the first 4 weeks and 15 mg/week for the subsequent 20 weeks	ACR20, ACR50, ACR70, ESR, CRP, RF, adverse events	a: 46.0 ± 10.6; b: 45.9 ± 10.4	47.2 ± 11.0	24 weeks
	Lu et al. (2009)	132	64	IGU 25 mg for the first 4 weeks and 50 mg for the subsequent 24 weeks	placebo	CRP, ESR, adverse events	57.5 ± 10.8	57.0 ± 10.8	28 weeks
	Xia et al. (2020)	50	50	IGU 25 mg Bid + MTX 7.5 mg once a week at the beginning, increase by 2.5 mg per week, with a final dose of 15 mg	MTX 7.5 mg once a week at the beginning, increase by 2.5 mg per week, with a final dose of 15 mg + Tripterygium glycosides 1–1.5 mg/kg	ESR, CRP	53.73 ± 2.78	53.62 ± 2.45	12 weeks
	Lu, 2014; Xia et al. (2016)	100	50	a: IGU 25 mg Bid + MTX 10 mg once a week; b: IGU 25 mg Bid	MTX 10 mg once a week	ESR, CRP	46.63 ± 10.61		24 weeks
	Zhao et al. (2016)	60	30	a: IGU 25 mg Bid + MTX 10 mg once a week; b: IGU 25 mg Bid	MTX 15 mg once a week	ACR20, ACR50, ACR70, adverse events	a: 30.1 ± 2.4; b: 29.3 ± 2.7	28.1 ± 3.4	24 weeks
	Shi et al. (2015)	30	30	IGU 25 mg Bid + MTX 10 mg once a week at the beginning; 12.5 mg twice a week after 4 weeks	MTX 10 mg once a week at the beginning; 12.5 mg twice a week after 4 weeks	DAS28, ESR, CRP, ACR20, ACR50, ACR70, adverse events	48.9 ± 12.2	48.4 ± 10.2	24 weeks
	Meng et al. (2015)	33	33	IGU 25 mg Bid + MTX 10 mg once a week	MTX 10 mg once a week + Leflunomide 10 mg Qd	DAS28, ACR20, ACR50, ACR70, adverse events	44.2 ± 20.5	41.7 ± 22.8	16 weeks
	Bi (2019)	30	30	IGU 25 mg Bid + MTX 10 mg once a week	MTX 10 mg once a week + Leflunomide 20 mg Qd	DAS28, adverse events	53.10 ± 12.90	54.60 ± 11.88	12 weeks
	Zhang (2018)	60	60	IGU 25 mg Qd	MTX 10 mg once a week + Leflunomide 20 mg Qd	ACR20, CRP, ESR, RF, adverse events	46.35 ± 18.19		24 weeks
	Li et al. (2016)	44	40	IGU 25 mg Qd + MTX 7.5–10 mg once a week	MTX 7.5–10 mg once a week + Tripterygium glycosides 20 mg Bid	DAS28, ESR, CRP, adverse events	60–77	60–82	12 weeks
	Mo et al. (2018)	30	30	IGU 25 mg Bid + MTX 10 mg once a week	MTX 10 mg once a week + Tripterygium glycosides 20 mg Bid	DAS28, ESR, CRP, CCP, RF, adverse events	45 ± 11.6	43.3 ± 10.25	12 weeks
	Duan et al. (2015)	30	30	IGU 25 mg Bid + MTX 10 mg once a week at the beginning, gradually increase to 12.5 mg within 4 weeks	MTX 10 mg once a week at the beginning, gradually increase to 12.5 mg within 4 weeks	ESR, CRP, DAS28, adverse events	48.9 ± 12.2	48.4 ± 10.2	24 weeks
	Xiong and GengGuanghui (2020)	51	51	IGU 25 mg Bid + MTX 10 mg once a week at the beginning; 12.5 mg twice a week after 2 weeks; 15 mg once a week after 4 weeks	MTX 10 mg once a week at the beginning; 12.5 mg twice a week after 2 weeks; 15 mg once a week after 4 weeks	Adverse events	48.21 ± 6.04	48.33 ± 5.93	24 weeks
	Shang (2014)	20	20	IGU 25 mg Bid	Etoricoxib 60 mg Qd	Adverse events	43.73 ± 3.62	45.73 ± 3.56	12 weeks
	Mo and Ma (2015)	30	30	IGU 25 mg Bid + MTX 15 mg once a week	MTX 15 mg once a week	ACR20, ACR50, ACR70, ESR, CRP, RF, adverse events	31.8 ± 8.5	31.9 ± 8.6	12 weeks
	Tian et al. (2020)	120	120	IGU 25 mg Bid + MTX 10 mg once a week	MTX 10 mg once a week + Leflunomide 20 mg Qd	DAS28, ESR, CRP, RF, adverse events	50 ± 10	49 ± 11	52 weeks
	Xu et al. (2015a)	72	38	a: IGU 25 mg Bid + MTX 7.5–20 mg once a week; b: IGU 25 mg Bid	MTX 7.5–20 mg once a week	ESR, CRP, RF, adverse events	a: 46.10 ± 17.09; b: 44.71 ± 9.32	43.28 ± 10.46	48 weeks
	Xu et al. (2017a)	42	41	IGU 25 mg Bid + MTX 7.5–20 mg once a week	MTX 7.5–20 mg once a week	DAS28, ESR, CRP	46.34 ± 2.29	46.19 ± 2.57	48 weeks
	Yan and Wang (2018)	35	35	IGU 25 mg Bid + MTX 10 mg once a week	MTX 10 mg once a week	Adverse events	56 ± 7	56 ± 7	24 weeks
	Fan et al. (2020)	38	37	IGU 25 mg Bid + MTX 10 mg once a week at the beginning; 12.5 mg once a week after 2 weeks; 15 mg once a week after 4 weeks	MTX 10 mg once a week at the beginning; 12.5 mg once a week after 2 weeks; 15 mg once a week after 4 weeks	DAS28	49.0 ± 10.1	48.7 ± 10.2	24 weeks
	Meng et al. (2016b)	30	30	IGU 25 mg Bid + MTX 15 mg once a week	MTX 15 mg once a week	DAS28, adverse events	41.6 ± 20.3	45.1 ± 19.2	16 weeks
	Wang et al. (2019a)	47	46	IGU 25 mg Bid + MTX 15 mg once a week	MTX 15 mg once a week	CRP, RF, ESR, DAS28	48.13 ± 6.40	47.83 ± 6.37	24 weeks
	Meng et al. (2017)	60	60	IGU 25 mg Bid + MTX 10 mg once a week	MTX 10 mg once a week	RF, CRP, adverse events	64.83 ± 9.41	64.31 ± 8.22	12 weeks
	Ju et al. (2020)	58	58	IGU 25 mg Bid + MTX 10 mg once a week	MTX 10 mg once a week	DAS28, ESR, CRP, RF	42.31 ± 13.78	41.87 ± 13.94	24 weeks
	Zhao and Hao (2018)	36	36	IGU 25 mg Bid + MTX 7.5 mg once a week	MTX 7.5 mg once a week	DAS28, CRP, adverse events	47.20 ± 3.40	50.80 ± 4.10	12 weeks
	Li and WH (2020)	20	13	IGU 25 mg Bid + MTX 10 mg once a week	MTX 10 mg once a week + Adalimumab 40 mg once every 2 weeks	DAS28	58 ± 11	55 ± 11	24 weeks
	Xu et al. (2015b)	30	28	IGU 25 mg Bid + MTX 10 mg once a week	MTX 10 mg once a week	RF, CRP, ESR, DAS28, adverse events	56 ± 12	51 ± 13	24 weeks
	Chen et al. (2018)	60	60	IGU 25 mg Bid + MTX 10 mg once a week	MTX 10 mg once a week	CRP, adverse events	45.7 ± 5.4	45.9 ± 4.8	24 weeks
	Zhao et al. (2017a)	63	33	a: IGU 25 mg Bid; b: IGU 25 mg Bid + MTX 10 mg once a week	MTX 10 mg once a week	ACR20, ACR50, ACR70, DAS28, ESR, CRP, RF, adverse events	a: 46.46 ± 11.01; b: 45.97 ± 10.75	46.31 ± 10.89	24 weeks
	Deng (2017)	59	31	a: IGU 25 mg Bid + MTX 10 mg once a week; b: IGU 25 mg Bid	MTX 10 mg once a week + Leflunomide 20 mg Qd	DAS28, ESR, CRP, RF, adverse events	47.23 ± 15.62		48 weeks
	Xie et al. (2018)	39	39	IGU 25 mg Bid + MTX 10 mg once a week at the beginning; 12.5 mg twice a week after 2 weeks; 15 mg once a week after 4 weeks	MTX 10 mg once a week at the beginning; 12.5 mg twice a week after 2 weeks; 15 mg once a week after 4 weeks	DAS28, adverse events	62.89 ± 4.57	62.74 ± 3.96	16 weeks
	Rao et al. (2014)	60	30	a: IGU 25 mg Bid; b: IGU 25 mg Qd	MTX 10 mg once a week	ACR20, ACR50, ACR70	42.6 ± 5.2		12 weeks
	Wang et al. (2022)	60	60	IGU 25 mg Bid + MTX 10 mg once a week	MTX 10 mg once a week	CRP, adverse events	54 ± 14	55 ± 13	12 weeks
	Dai et al. (2022)	60	60	IGU 25 mg Bid + MTX 7.5 mg once a week	MTX 7.5 mg once a week	DAS28, CRP, ESR, RF	59.4 ± 7.8	60.1 ± 9.7	12 weeks
	Sun and Li (2022)	43	43	IGU 25 mg Bid + MTX 10 mg once a week	MTX 10 mg once a week	Adverse events	49.05 ± 4.32	48.96 ± 5.24	24 weeks
	Wu et al. (2022)	58	58	IGU 25 mg Bid + MTX 10 mg once a week + Tripterygium wilfordii polyglycosides 50 mg for the first time and 20 mg Qd after 3days	MTX 10 mg once a week + Tripterygium wilfordii polyglycosides 50 mg for the first time and 20 mg Qd after 3days	DAS28, CRP, ESR, RF	61.48 ± 4.36	62.73 ± 4.58	18 weeks
	DongZhang et al. (2019)	52	104	IGU 25 mg Bid + Tripterygium glycosides 1.5 mg/(kg·d)	a: Prednisone + Sulfasalazine; b: Tripterygium glycosides 1.5 mg/(kg·d)	Forced vital capacity (FVC), Forced expiratory volume in 1 s (FEV1), total lung capacity (TLC), CRP, RF, adverse events	54.7 ± 5.1	a: 55.6 ± 4.9; b: 54.1 ± 5.4	24 weeks
AS	Qiu et al. (2016)	18	18	Iguratimod 25 mg Bid	NSAIDs + DMARDs	ESR, Bath Ankylosing Spondylitis Disease Activity Index (BASDAI), Bath Ankylosing Spondylitis Functional Index (BASFI), visual analogue scale (VAS), back pain score, adverse events	37.3 ± 7.0	34.5 ± 9.3	24 weeks
	Yuan et al. (2020)	41	39	Iguratimod 25 mg Bid + Etoricoxib tablets 60 mg Qd. + ibuprofen 300 mg Tid. + methotrexate 15 mg once a week	Etoricoxib tablets 60 mg Qd. + ibuprofen 300 mg Tid. + methotrexate 15 mg once a week	VAS, CRP, ESR, adverse events	39.28 ± 5.30	40.08 ± 5.67	12 weeks
	Pang et al. (2020)	39	39	Iguratimod 25 mg Bid + Etanercept 25 mg tiwce a week	Etanercept 25 mg tiwce a week	ESR, CRP, BASDAI	24.85 ± 4.18	25.01 ± 4.29	12 weeks
	Lin et al. (2019)	24	24	Iguratimod 25 mg Bid + Sulfasalazine 1 g Bid. + methotrexate 10 mg once a week + NSAIDs	Sulfasalazine 1 g Bid. + methotrexate 10 mg once a week + NSAIDs	BASDAI, BASFI, VAS, adverse events	32.71 ± 8.80	28.21 ± 6.69	24 weeks
	Xu et al. (2019)	21	21	Iguratimod 25 mg Bid + Celecoxib 0.2 g Qd	Sulfasalazine 1 g Bid. + Celecoxib 0.2 g Qd	BASDAI, BASFI, VAS, ESR, CRP, adverse events	35.1 ± 10.3	34.3 ± 9.5	24 weeks
	Zeng et al. (2016)	25	25	Iguratimod 25 mg Bid + Meloxicam 7.5 mg Qd	Sulfasalazine 0.75 g Tid. + Meloxicam 7.5 mg Qd	BASDAI, CRP, adverse events	38 ± 12	40 ± 10	24 weeks
	Li et al. (2021a)	48	25	Iguratimod 50 mg Qd + NSAIDs	NSAIDs + Placebo	BASDAI, BASFI, CRP, ESR, adverse events	31.38 ± 7.36	30.28 ± 5.94	24 weeks
	Bai et al. (2021)	43	43	Iguratimod 25 mg Bid + Sulfasalazine 1 g Bid + Celecoxib 200 mg Bid	Sulfasalazine 1 g Bid + Celecoxib 200 mg Bid	BASDAI, VAS, CRP, ESR, adverse events	28.52 ± 9.43	27.87 ± 8.05	12 weeks
	Li et al. (2021b)	30	30	Iguratimod 25 mg Bid + Sulfasalazine 0.5–1 g Bid + Thalidomide 50–200 mg Qn	Sulfasalazine 0.5–1 g Bid + Thalidomide 50–200 mg Qn	BASDAI	31.24 ± 4.71	30.01 ± 4.68	24 weeks
PS	Gu (2020)	40	40	Iguratimod 25 mg Bid	Prednisone 8 mg Qd + HCQ 200 mg Bid	RF, Adverse events	66.72 ± 4.34	66.51 ± 4.23	12 weeks
	Jiang et al. (2014)	25	25	Iguratimod 25 mg Bid	Prednisone 5–10 mg Qd + HCQ 200 mg Bid + Bromoethylsine 16 mg Bid	EULAR SS Patient Reported Index (ESSPRI), EULAR SS disease activity index (ESSDAI), Schirmer’s test, Adverse events	29.3 ± 9.7	32.5 ± 11.5	12 weeks
	Zhao (2019)	41	41	Iguratimod 25 mg Bid	Prednisone 8 mg Qd + HCQ 200 mg Bid	RF, ESR, Adverse events	55.51 ± 6.52	54.52 ± 6.54	12 weeks
	Lu and Zhang (2021)	48	48	Iguratimod 25 mg Bid + HCQ 0.2 g Bid	HCQ 0.2 g Bid	ESR, RF, adverse events	45.52 ± 7.48	44.24 ± 8.32	12 weeks
	Li et al. (2020)	23	23	Iguratimod 25 mg Bid	Prednisone 8 mg Qd + HCQ 200 mg Bid	ESSPRI, ESR, Adverse events	46.29 ± 1.24	46.38 ± 1.37	12 weeks
	Zhang (2019)	60	60	Iguratimod 25 mg Bid + Methylprednisolone 8 mg	Methylprednisolone 8 mg Qd + HCQ 200 mg Bid	ESSPRI, ESSDAI, Schirmer’s test	49.43 ± 3.74		12 weeks
	Jia (2020)	43	43	Iguratimod 25 mg Bid	Methylprednisolone 8 mg Qd + HCQ 200 mg Bid	ESSPRI, ESSDAI, ESR, RF, adverse events	50.47 ± 9.11	50.47 ± 9.11	16 weeks
	Yu (2020)	38	38	Iguratimod 25 mg Bid	Methylprednisolone 8 mg Qd + HCQ 200 mg Bid	ESR, RF	41.18 ± 3.36	41.14 ± 3.39	12 weeks
	Shao et al. (2020)	44	22	Iguratimod 25 mg Bid	Placebo	ESSPRI, ESR, ESSDAI, Adverse events	49.5 ± 12.3	48.2 ± 11.5	24 weeks
	Chen et al. (2022)	62	62	Iguratimod 25 mg Bid + Total Glucosides of Paeony 0.6 g Tid + HCQ 0.2 g Bid	Total Glucosides of Paeony 0.6 g Tid + HCQ 0.2 g Bid	ESSPRI, ESSDAI, ESR, RF	68.02 ± 3.02	68.50 ± 3.05	12 weeks
	Donghui (2019)	30	30	Iguratimod 25 mg Bid + Methylprednisolone 8 mg	Methylprednisolone 8 mg Qd + HCQ 200 mg Bid	ESR, adverse events	46.9 ± 4.2	46.5 ± 4.3	12 weeks
	Zhang and Shen (2019)	43	43	Iguratimod 25 mg Bid + Methylprednisolone 8 mg	Methylprednisolone 8 mg Qd + HCQ 200 mg Bid	ESSPRI, ESSDAI, ESR, RF, Schirmer’s test, adverse events	40.35 ± 9.41	41.03 ± 10.01	12 weeks
	Jiang et al. (2016)	30	30	Iguratimod 50 mg Qd	Prednisone 8 mg Qd + HCQ 200 mg Bid	RF, ESR, Adverse events	45.13 ± 12.11	46.33 ± 13.74	12 weeks
	Xie et al. (2020b)	38	38	Iguratimod 25 mg Bid + Total Glucosides of Paeony 0.6 g Tid + HCQ 0.2 g Bid	Total Glucosides of Paeony 0.6 g Tid + HCQ 0.2 g Bid	ESR, CRP, Schirmer’s test, Adverse events	57.3 ± 7.92	56.8 ± 8.44	24 weeks
	Jiang et al. (2020)	25	25	Iguratimod 50 mg Qd	Prednisone 10 mg, hydroxychloroquine (HCQ) 400 mg, new hydrochloride bromine ethyl Qd	EULAR Sjögren’s syndrome patient-reported index (ESSPRI), ESSDAI, Schirmer’s test, Adverse events	29.3 ± 9.7	32.5 ± 11.5	12 weeks
	Bai and Jiao (2019)	30	30	Iguratimod 25 mg Bid	Methylprednisolone 8 mg Qd + HCQ 200 mg Bid + Leflunomide 50 mg Qd	ESSPRI, ESSDAI, RF, ESR, Adverse events	43 ± 21	43 ± 10	12 weeks
	Rao et al. (2022)	43	43	Iguratimod 25 mg Bid	Methylprednisolone 4 mg Qd + HCQ 200 mg Bid	Schirmer’s test, ESR, RF	51.8 ± 10.3	50.1 ± 9.9	12 weeks
	Ding et al. (2022)	20	20	Iguratimod 25 mg Bid + HCQ 100 mg Bid + Prednisone 5 mg Bid	HCQ 100 mg Bid + Prednisone 5 mg Bid	ESSPRI, ESSDAI, ESR, RF, Schirmer’s test, adverse events	66.15 ± 3.71	66.31 ± 3.98	12 weeks
	Xu et al. (2017b)	47	47	Iguratimod 25 mg Bid	Prednisone 8 mg Qd + HCQ 200 mg Bid	ESSPRI, ESSDAI, ESR, RF, Schirmer’s test	44.5 ± 13.2	45.3 ± 13.1	12 weeks
	Zhang et al. (2019)	100	100	Iguratimod 25 mg Bid	Prednisone + HCQ + olfaction	FVC, maximum mid-expiratory flow (MMF), ESR, adverse events	30.68 ± 3.51	31.00 ± 3.60	20 weeks
	Luo et al. (2018b)	40	40	Iguratimod 25 mg Bid	Prednisone 8 mg Qd + HCQ 200 mg Bid	ESR, RF, adverse events	43.6 ± 10.5	45.2 ± 12.9	12 weeks
	Wang et al. (2019b)	32	32	Iguratimod 25 mg Bid + Total Glucosides of Paeony 0.6 g Bid + HCQ 0.1 g Bid	Total Glucosides of Paeony 0.6 g Bid + HCQ 0.1 g Bid	ESSPRI, ESSDAI, Schirmer’s test, ESR, RF, Adverse events	66.8 ± 7.7	65.3 ± 8.2	12 weeks
	Zhao (2020)	25	25	Iguratimod 25 mg Bid + Basic therapy	HCQ 200 mg Bid + Basic therapy	ESR, RF, adverse events	45.3 ± 2.8	45.7 ± 2.8	Unkown
	Liang et al. (2021)	30	30	Iguratimod 25 mg Bid + Methylprednisolone 8 mg	Methylprednisolone 8 mg Qd + HCQ 200 mg Bid	ESSDAI, ESSPRI, ESR, CRP, adverse events	45.16 ± 6.37	40.15 ± 6.65	16 weeks
	Li et al. (2018)	34	34	Iguratimod 25 mg Bid	Prednisone 8 mg Qd + HCQ 200 mg Bid	ESSPRI, RF, ESR, Adverse events	40.05 ± 3.16	40.02 ± 3.15	12 weeks
	Jiang (2021)	24	22	Iguratimod 25 mg Bid + Chere Cunjing Granules	Chere Cunjing Granules (Traditional Chinese Medicine)	ESSPRI, ESSDAI, ESR, CRP, adverse events	45.95 ± 11.52	48.92 ± 11.53	12 weeks
	Yi (2018)	20	20	Iguratimod 25 mg Bid + Total Glucosides of Paeony 0.6 g Tid + HCQ 0.2 g Bid	Total Glucosides of Paeony 0.6 g Tid + HCQ 0.2 g Bid	ESR, CRP, Adverse events	56.87 ± 2.56	56.23 ± 2.86	12 weeks
	Zhuang (2020)	34	34	Iguratimod 25 mg Bid + Methylprednisolone 8 mg	Methylprednisolone 8 mg Qd + HCQ 200 mg Bid	ESR, RF	36.48 ± 1.25	36.51 ± 1.19	12 weeks
	Xia et al. (2017)	50	50	Iguratimod 25 mg Bid + Methylprednisolone	HCQ 200 mg Bid + Methylprednisolone	ESR, RF	42.13 ± 9.97	42.08 ± 9.65	12 weeks
	Gu (2022)	42	42	Iguratimod 25 mg Bid	Methylprednisolone 8 mg Qd + HCQ 200 mg Bid	ESSPRI, adverse events	40.97 ± 10.24	41.56 ± 10.21	2 weeks
	Liu (2022)	40	40	Iguratimod 25 mg Bid + Total Glucosides of Paeony 0.6 g Tid + HCQ 200 mg Bid + methylprednisolone 8 mg Qd	Total Glucosides of Paeony 0.6 g Tid + HCQ 200 mg Bid + methylprednisolone 8 mg Qd	ESSDAI, ESSPRI, ESR, RF, adverse events	44.05 ± 8.82	43.68 ± 8.75	12 weeks
	Zhuang et al. (2021)	10	10	Iguratimod 25 mg Bid + Prednisone 5–10 mg Tid	Cyclophosphamide + Prednisone 5–10 mg Tid	Dispersive carbon monoxide (DLCO), 6-min walk test (6MWT), CRP, ESR, RF, adverse events	45.69 ± 2.80	45.31 ± 2.78	24 weeks

### 3.3 Risk of bias assessments

**FIGURE 2:**
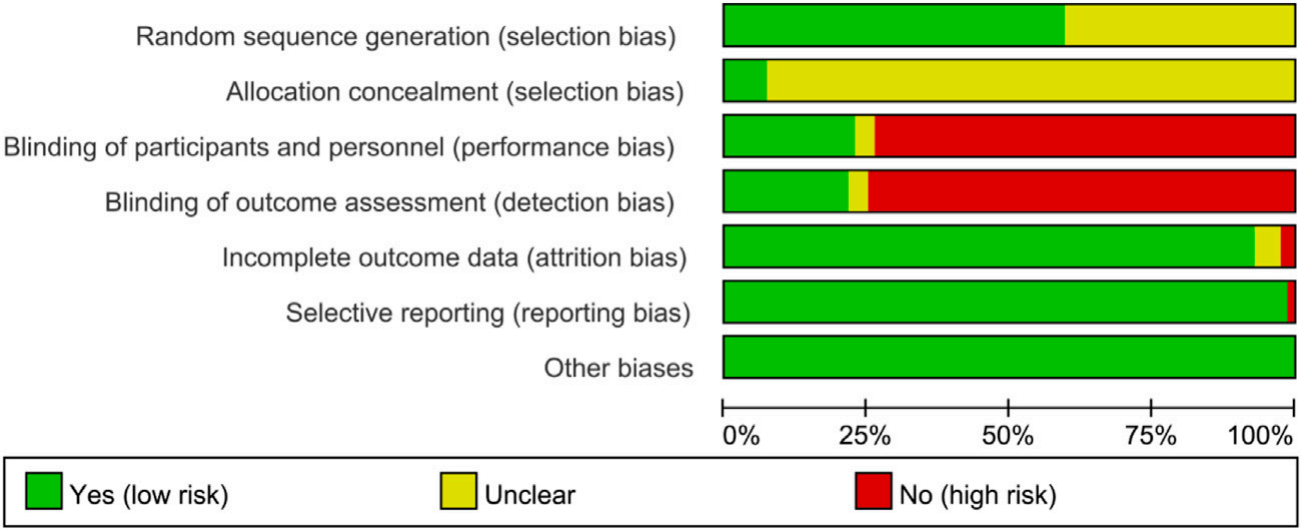
Risk of bias graph.

**FIGURE 3:**
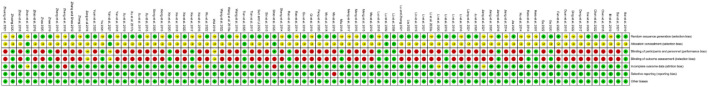
Risk of bias summary.

The summary and graph of risk of bias ware shown in Figures 2, 3.

#### 3.3.1 Sequence generation and allocation concealment

Fifty RCTs described detailed random sequence generation methods and were therefore assessed as low risk of bias, whereas the remainder were assessed as unclear risk of bias. Lü et al. (2008), Du et al. (2008), Tian et al. (2020), Zhao et al. (2017a), Li Y. et al., 2021) and Shao et al. (2020) described methods of allocation concealment and was therefore assessed as low risk of bias, whereas the remainder were assessed as unclear risk of bias.

#### 3.3.2 Blinding

Zeng et al. (2016), Li Y. et al. (2021), and Donghui (2019) reported the use of blinding in their RCTs, but did not provide sufficient details about the implementation process, resulting in an unclear risk of bias assessment. Of the total 84 RCTs, 19 reported blinding of participants, and 18 reported blinding of assessors, indicating a low risk of bias. The remaining RCTs were assessed as high risk of bias because blinding was not described and outcomes included subjectively assessed outcomes.

#### 3.3.3 Incomplete outcome data and selective reporting

Zhang (2018) and SShao et al. (2020) had incomplete outcomes and were therefore assessed as high risk of bias. There was not enough evidence to prove whether there were incomplete outcomes in Lu (2014), Xia et al. (2016), Li and WH (2020), Zhao et al. (2017a) and Jiang (2021), so they were assessed as unknown risk of bias. The remaining RCTs did not have incomplete outcomes and were therefore assessed as low risk of bias.

Mo et al. (2018) did not report all data planned in the methodology and was therefore assessed as high risk of bias. The remaining RCTs did not have selective reports and were therefore assessed as low risk of bias.

#### 3.3.4 Other potential bias

No other sources of bias were identified in any of the RCTs, indicating a low risk of bias from other sources.

### 3.4 IGU for RA

#### 3.4.1 RA remission rate

ACR20, ACR50 and ACR70 were used to represent RA remission rate. According to the medication of the IGU group, it is divided into IGU + MTX subgroup and IGU only subgroup.

**FIGURE 4:**
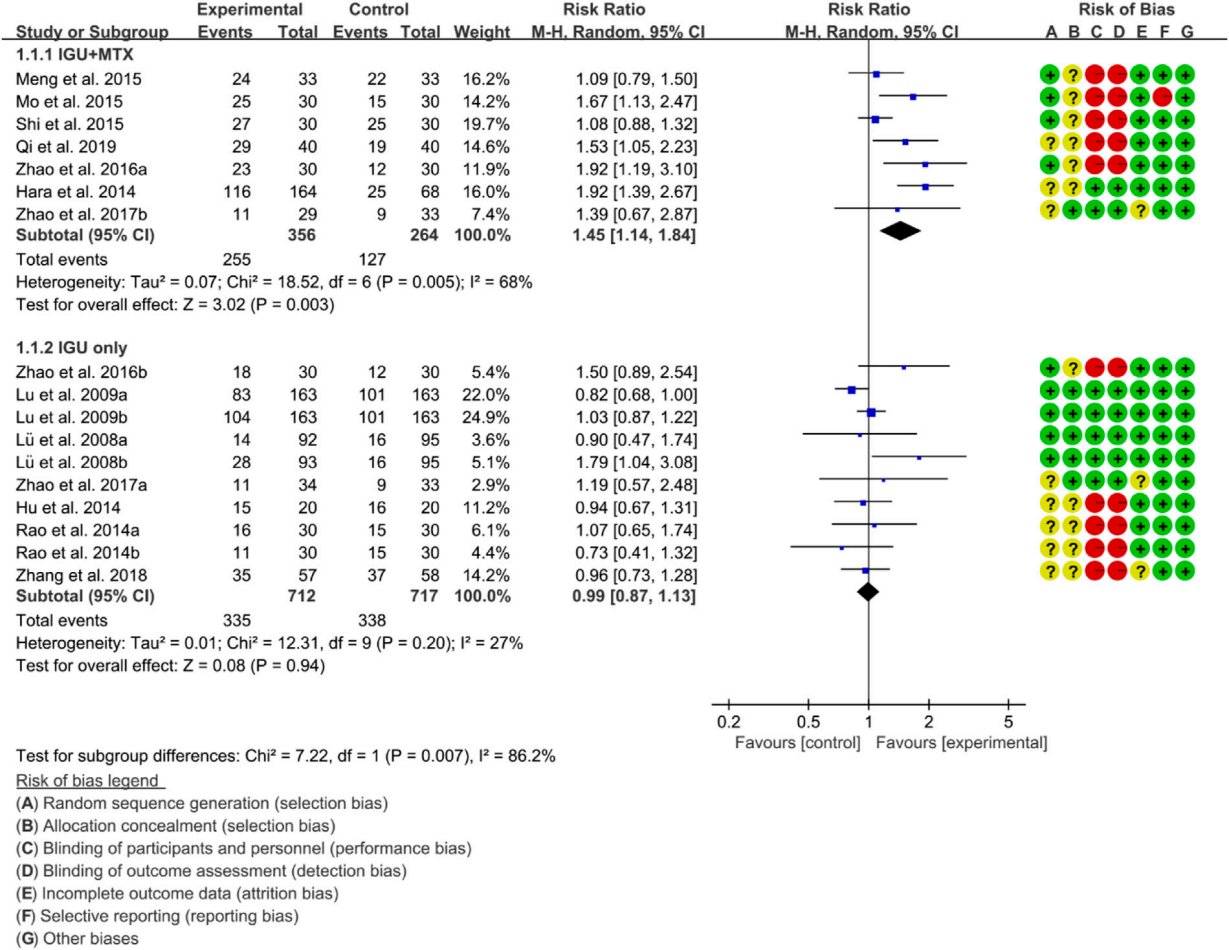
ACR20.

For ACR20, the heterogeneity test showed that some subgroups had high heterogeneity (IGU + MTX subgroup: *p* = 0.005, I2 = 68%; IGU only subgroup: *p* = 0.20, I2 = 27%), and a random effect model was used. The meta-analysis findings indicate that the IGU + MTX group had a significantly lower ACR20 compared to the control group (RR 1.45 [1.14, 1.84], *p* = 0.003; random-effect model). However, there was no significant difference in ACR20 between the IGU-only group and the control group (RR 0.99 [0.87, 1.13], *p* = 0.94; random-effect model) (Figure 4). The results of publication bias test showed that it was less likely to have publication bias in IGU + MTX subgroup (*p* = 0.313) and IGU only subgroup (*p* = 0.396).

**FIGURE 5:**
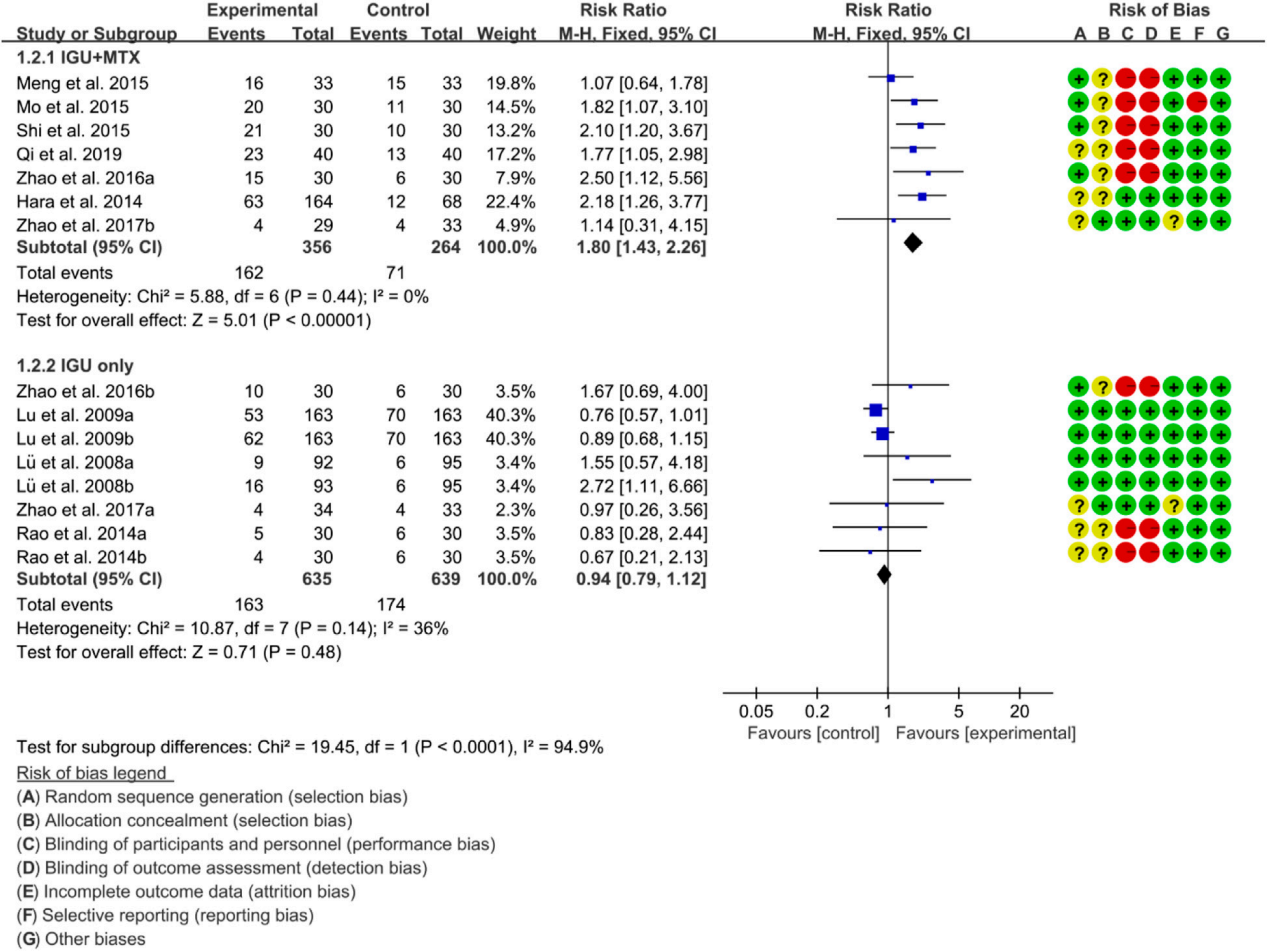
ACR50.

For ACR50, the heterogeneity test showed that the heterogeneity was low (IGU + MTX subgroup: *p* = 0.44, I2 = 0%; IGU only subgroup: *p* = 0.14, I2 = 36%), and a fixed effect model was used. The meta-analysis findings indicate that the IGU + MTX group had a lower ACR50 compared to the control group (RR 1.80 [1.43, 2.26], *p* < 0.00001; fixed-effect model). However, there was no significant difference between the IGU only group and the control group (RR 0.94 [0.79, 1.12], *p* = 0.48; fixed-effect model) (Figure 5). The results of publication bias test showed that it was less likely to have publication bias in IGU + MTX subgroup (*p* = 0.433) and IGU only subgroup (*p* = 0.245).

**FIGURE 6:**
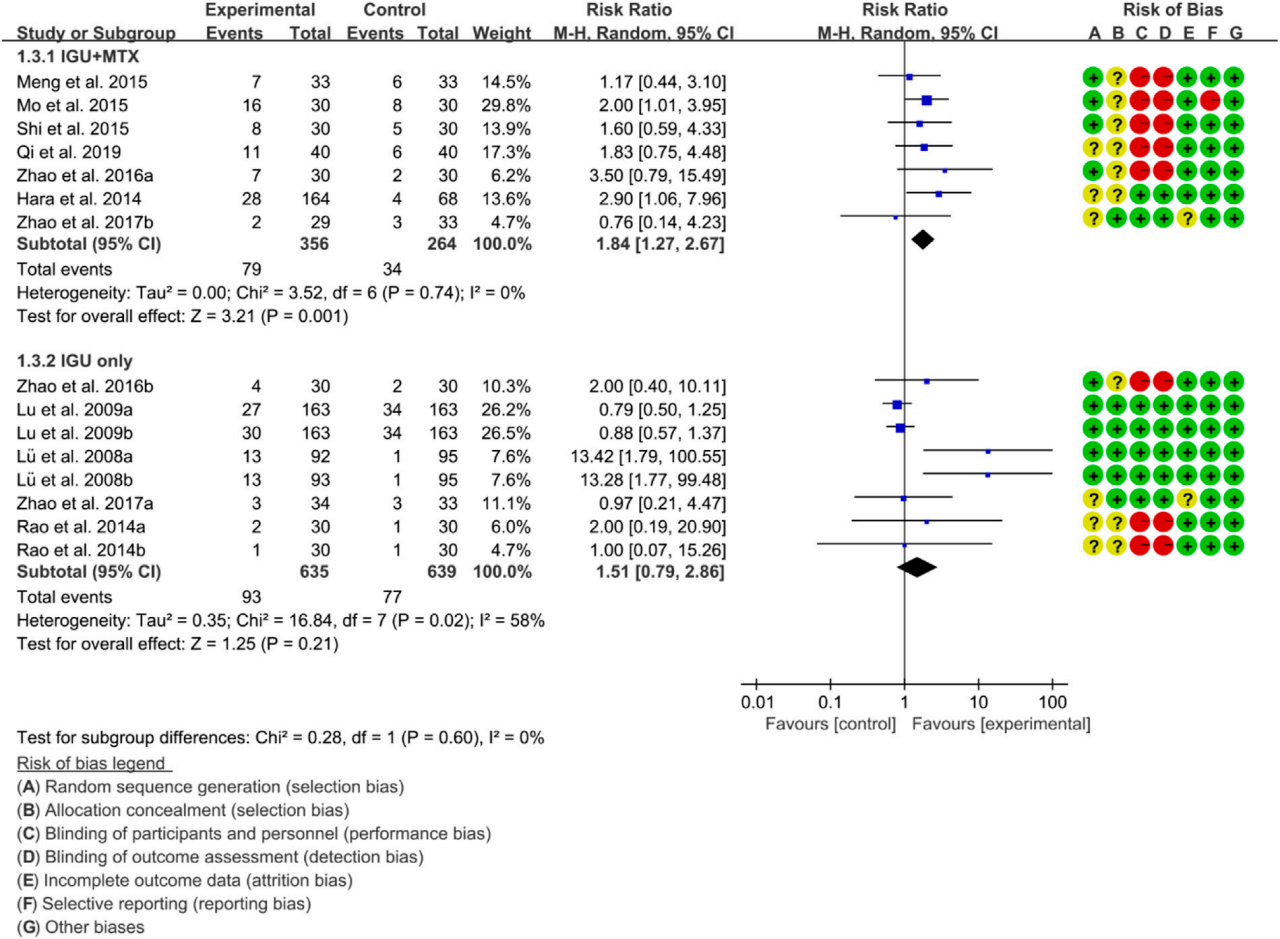
ACR70.

For ACR70, the heterogeneity test showed that some subgroups had high heterogeneity (IGU + MTX subgroup: *p* = 0.74, I2 = 0%; IGU only subgroup: *p* = 0.02, I2 = 58%), and a random effect model was used. The findings of the meta-analysis indicate that the IGU + MTX group had a lower ACR70 than the control group (RR 1.84 [1.27, 2.67], *p* = 0.001; random effect model), while the difference between the IGU only group and the control group did not reach statistical significance (RR 1.51 [0.79, 2.86], *p* = 0.21; random effect model) (Figure 6). The results of publication bias test showed that it was less likely to have publication bias in IGU + MTX subgroup (*p* = 0.193) and IGU only subgroup (*p* = 0.230).

#### 3.4.2 DAS28

**FIGURE 7:**
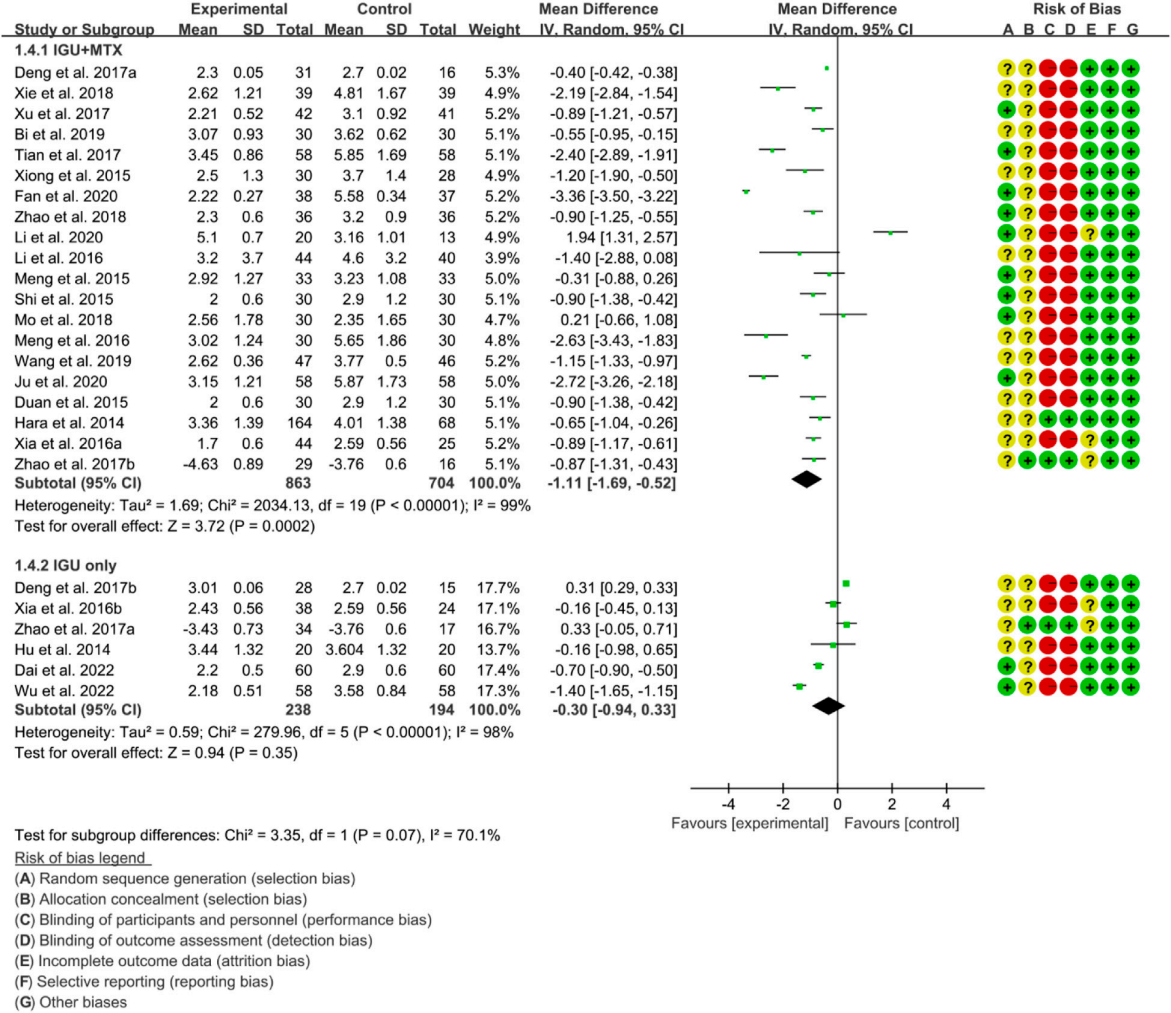
DAS28.

According to the medication of the IGU group, it is divided into IGU + MTX subgroup and IGU only subgroup. The heterogeneity test showed that the heterogeneity was high (IGU + MTX subgroup: *p* < 0.00001, I2 = 99%; IGU only subgroup: *p* < 0.00001, I2 = 98%), and a random effect model was used. According to the meta-analysis results, the IGU + MTX group showed a significant decrease in DAS28 compared to the control group (WMD −1.11 [−1.69, −0.52], *p* = 0.0002; random effect model). However, the difference between the IGU only group and control group was not statistically significant (WMD −0.30 [−0.94, 0.33], *p* = 0.35; random effect model) (Figure 7). The results of publication bias test showed that it may be likely to have publication bias in IGU + MTX subgroup (*p* = 0.080); but was less likely in and IGU only subgroup (*p* = 0.122).

#### 3.4.3 Inflammatory factor

Inflammatory factors include CRP, ESR and RF. According to the medication of the IGU group, it is divided into IGU + MTX subgroup, IGU only subgroup and IGU + Tripterygium Extract subgroup.

**FIGURE 8:**
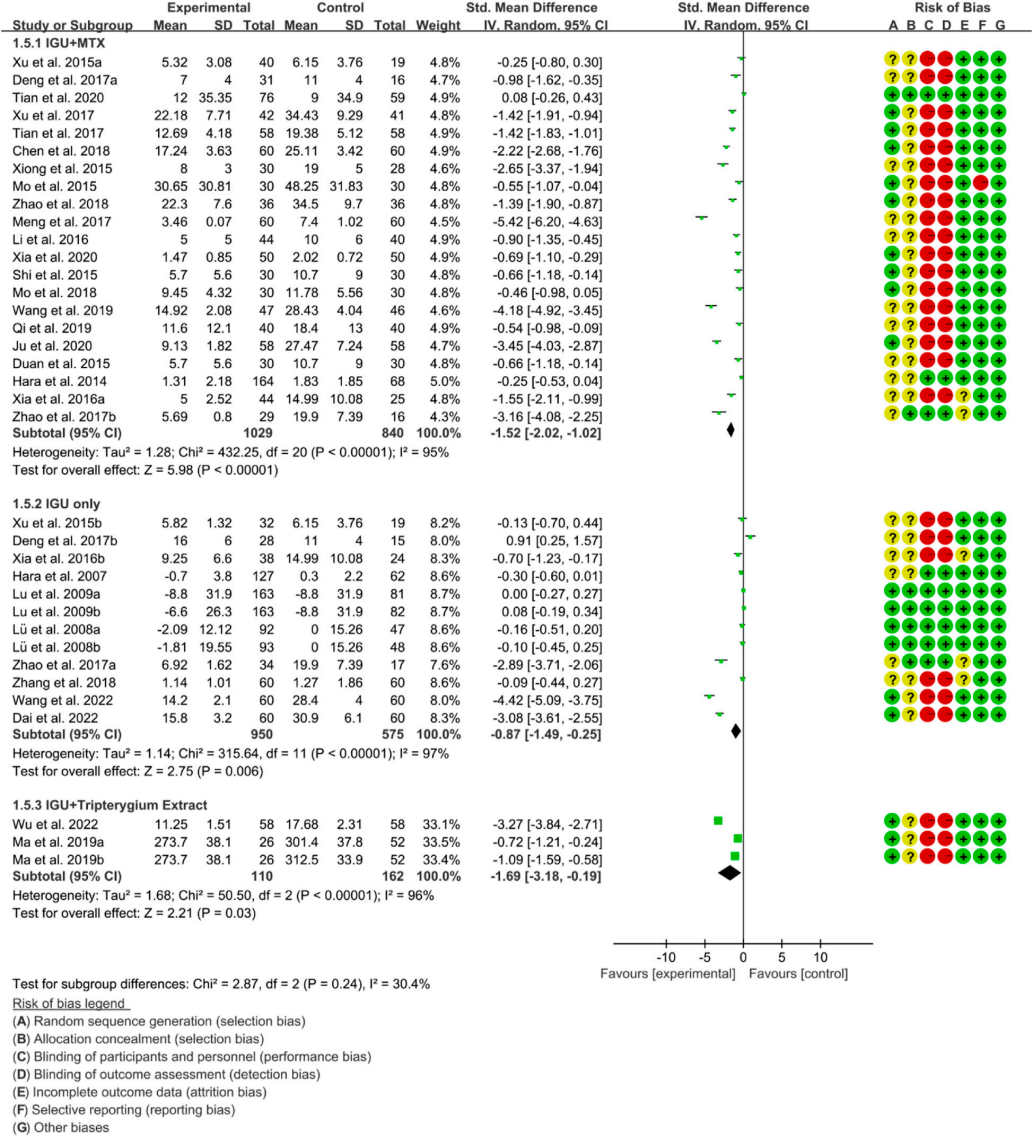
Crp.

For CRP, the heterogeneity test showed that the heterogeneity was high (IGU + MTX subgroup: *p* < 0.00001, I2 = 95%; IGU only subgroup: *p* < 0.00001, I2 = 96%; IGU + Tripterygium Extract subgroup: *p* < 0.00001, I2 = 96%), and a random effect model was used. The meta-analysis results show that compared with the control group, the CRP in the IGU + MTX group, IGU only subgroup and IGU + Tripterygium Extract subgroup was lower (Figure 8).

**FIGURE 9:**
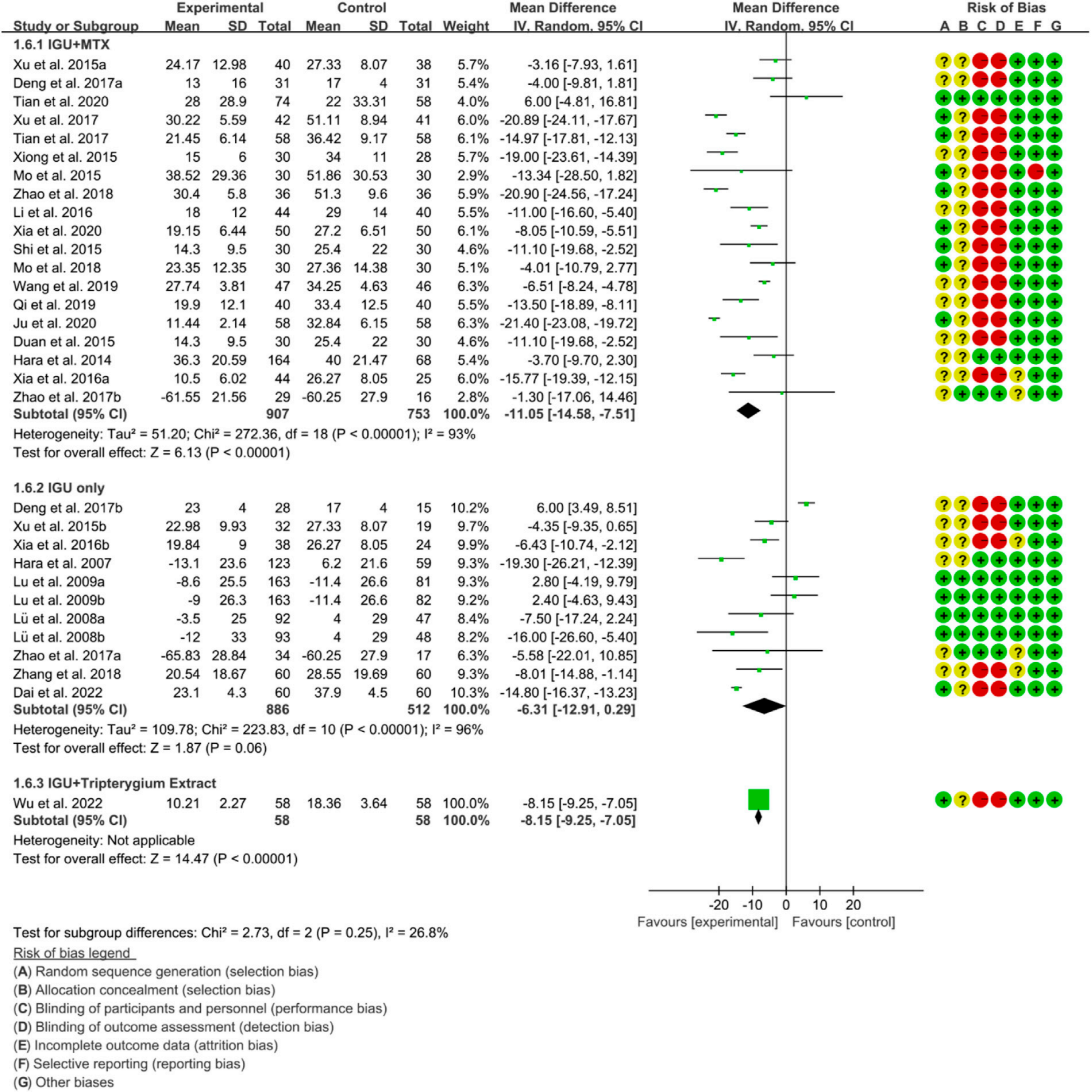
Esr.

For ESR, the heterogeneity test showed that the heterogeneity was high (IGU + MTX subgroup: *p* < 0.00001, I2 = 93%; IGU only subgroup: *p* < 0.00001, I2 = 96%; IGU + Tripterygium Extract subgroup: *p* < 0.00001, I2 = 96%), and a random effect model was used. The meta-analysis results show that compared with the control group, the ESR in the IGU + MTX group (WMD −11.05 [−14.58, −7.51], *p* < 0.00001; random effect model) and IGU + Tripterygium Extract group was lower (WMD −8.15 [−9.25, −7.05], *p* < 0.00001; random effect model), while its difference between IGU only group and control group was of no statistical significance (WMD −6.31 [−12.91, 0.29], *p* = 0.06; random effect model) (Figure 9).

**FIGURE 10:**
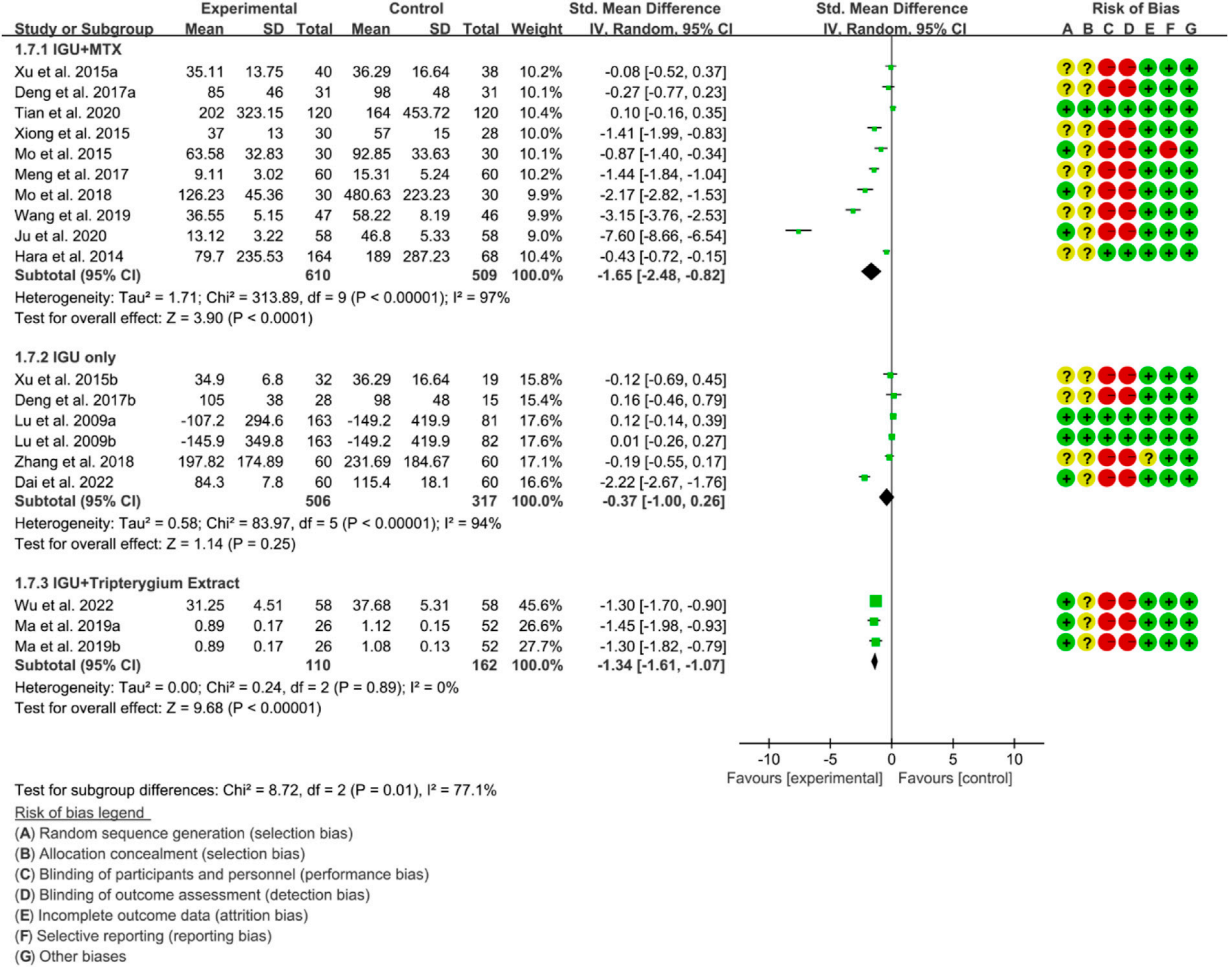
Rf.

For RF, the heterogeneity test showed that the heterogeneity was high (IGU + MTX subgroup: *p* < 0.00001, I2 = 97%; IGU only subgroup: *p* < 0.00001, I2 = 94%; IGU + Tripterygium Extract subgroup: *p* = 0.89, I2 = 0%), and a random effect model was used. The meta-analysis results indicate that compared with the control group, the RF in the IGU + MTX group (SMD −1.65 [−2.48, −0.82], *p* < 0.0001; random effect model) and IGU + Tripterygium Extract group were significantly lower (SMD −1.34 [−1.61, −1.07], *p* < 0.00001; random effect model). However, there was no significant difference between the IGU only group and control group (SMD −0.37 [−1.00, 0.26], *p* = 0.25; random effect model) (Figure 10).

#### 3.4.4 Adverse events

**FIGURE 11:**
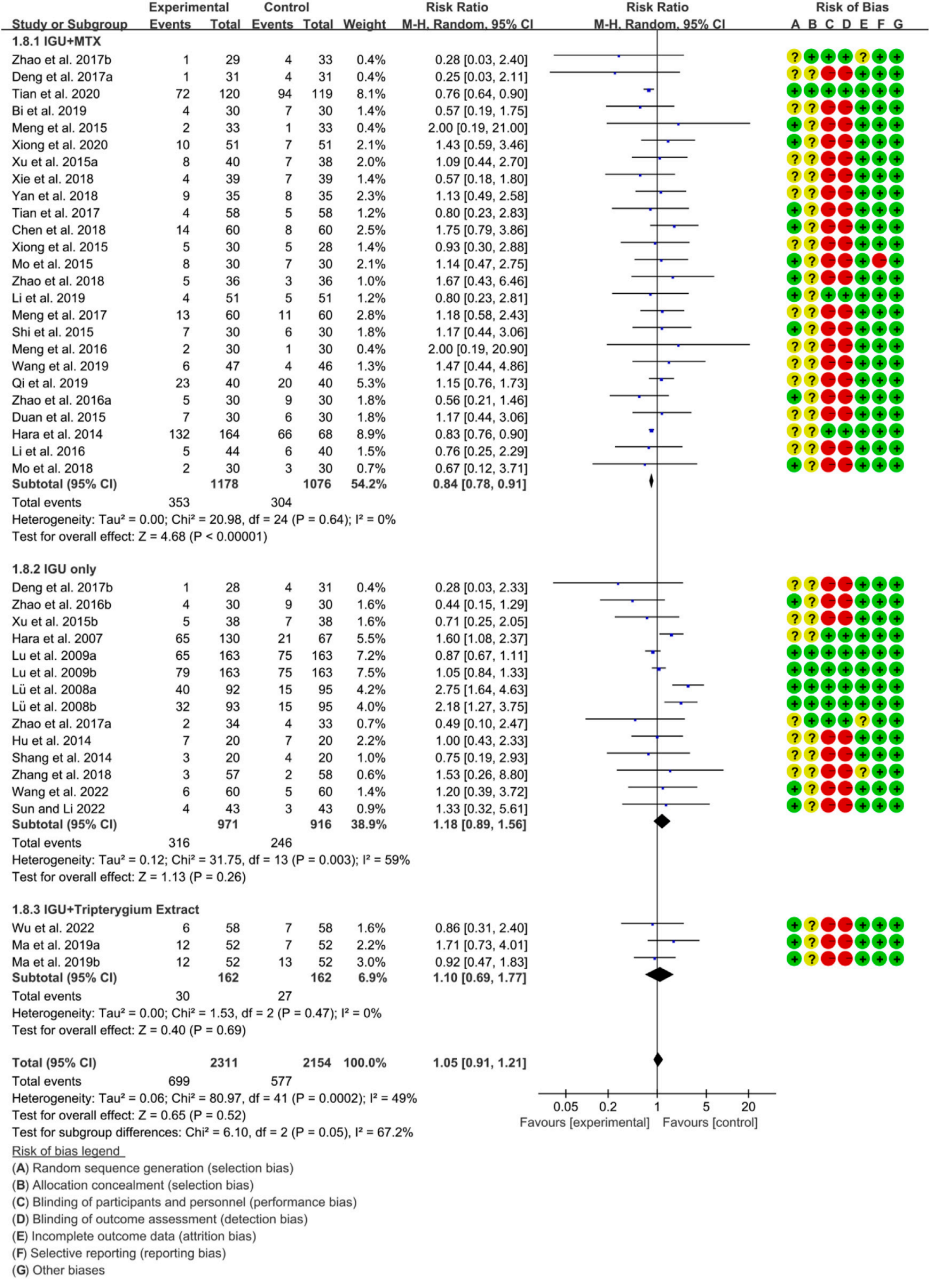
Adverse events for RA.

According to the medication of the IGU group, it is divided into IGU + MTX subgroup, IGU only subgroup and IGU + Tripterygium Extract subgroup. The heterogeneity test showed that the heterogeneity was high (IGU + MTX subgroup: *p* = 0.64, I2 = 0%; IGU only subgroup: *p* = 0.003, I2 = 59%; IGU + Tripterygium Extract subgroup: *p* = 0.47, I2 = 0%), and a random effect model was used. The meta-analysis results show that compared with the control group, the adverse events in the IGU + MTX group was lower (RR 0.84 [0.78, 0.91], *p* < 0.00001; random effect model), while its difference between IGU only group and control group (RR 1.18 [0.89, 1.56], *p* = 0.26; random effect model), and between IGU + Tripterygium Extract and control group was of no statistical significance (RR 1.10 [0.69, 1.77], *p* = 0.69; random effect model) (Figure 11). The results of publication bias test showed that it was less likely to have publication bias in IGU + MTX subgroup (*p* = 0.443) and in IGU only subgroup (*p* = 0.474).

#### 3.4.5 Quality of evidence

Only IGU + MTX and IGU only subgroups met the requirements of publication bias detection and evidence quality assessments.

According to the GRADE handbook, the evidence of IGU + MTX subgroup was judged to be moderate to very low (Table 2). The evidence of IGU only subgroup was judged to be moderate to low (Table 3).

**TABLE 2:** Evidence quality of IGU for RA in IGU + MTX subgroup.

Outcomes	Illustrative comparative risks* (95% CI)		Relative effect (95% CI)	No of participants (studies)	Quality of the evidence (GRADE)	Comments
	Assumed risk	Corresponding risk				
	Control					
ACR20 - IGU + MTX	Study population		RR 1.45 (1.14–1.84)	620 (7 studies)	⊕⊕⊝⊝ low^a^ ^,^ ^b^	
	481 per 1,000	698 per 1,000 (548–885)				
	Moderate					
	475 per 1,000	689 per 1,000(541–874)				
ACR50 - IGU + MTX	Study population		RR 1.8 (1.43–2.26)	620 (7 studies)	⊕⊕⊕⊝ moderate^a^ ^,^ ^b^	
	269 per 1,000	484 per 1,000 (385–608)				
	Moderate					
	325 per 1,000	585 per 1,000 (465–734)				
ACR70 - IGU + MTX	Study population		RR 1.84 (1.27–2.67)	620 (7 studies)	⊕⊕⊕⊝ moderate^a^ ^,^ ^b^	
	129 per 1,000	237 per 1,000 (164–344)				
	Moderate					
	150 per 1,000	276 per 1,000 (190–401)				
DAS28 - IGU + MTX		The mean DAS28-IGU + MTX in the intervention groups was 1.11 lower (1.69–0.52 lower)		1,567 (20 studies)	⊕⊝⊝⊝ very low^a^ ^,^ ^b^ ^,^ ^c^	
AEs - IGU + MTX	Study population		RR 0.84 (0.78–0.91)	2,254 (25 studies)	⊕⊕⊕⊝ moderate^a^	
	283 per 1,000	237 per 1,000 (220–257)				
	Moderate					
	179 per 1,000	150 per 1,000 (140–163)				

*The basis for the assumed risk (e.g., the median control group risk across studies) is provided in footnotes. The corresponding risk (and its 95% confidence interval) is based on the assumed risk in the comparison group and the relative effect of the intervention (and its 95% CI).

CI: confidence interval; RR: Risk ratio.

GRADE, working group grades of evidence.

High quality: Further research is very unlikely to change our confidence in the estimate of effect.

Moderate quality: Further research is likely to have an important impact on our confidence in the estimate of effect and may change the estimate.

Low quality: Further research is very likely to have an important impact on our confidence in the estimate of effect and is likely to change the estimate.

Very low quality: We are very uncertain about the estimate.

^a^ Downgraded one level due to serious risk of bias (random sequence generation, allocation concealment, blinding, incomplete outcomes) and most of the data comes from the RCTs, with moderate risk of bias.

^b^ Downgraded one level due to the probably substantial heterogeneity.

^c^ Downgraded one level due to potential publication bias.

**TABLE 3:** Evidence quality of IGU for RA in IGU only subgroup.

Outcomes	Illustrative comparative risks* (95% CI)		Relative effect (95% CI)	No of participants (studies)	Quality of the evidence (GRADE)	Comments
	Assumed risk	Corresponding risk				
	Control					
ACR20 - IGU only	Study population		RR 0.99 (0.87–1.13)	1,429 (10 studies)	⊕⊕⊕⊝ moderate^a^	
	471 per 1,000	467 per 1,000 (410–533)				
	Moderate					
	500 per 1,000	495 per 1,000 (435–565)				
ACR50 - IGU only	Study population		RR 0.94 (0.79–1.12)	1,274 (8 studies)	⊕⊕⊕⊝ moderate^a^	
	272 per 1,000	256 per 1,000 (215–305)				
	Moderate					
	200 per 1,000	188 per 1,000 (158–224)				
ACR70 - IGU only	Study population		RR 1.51 (0.79–2.86)	1,274 (8 studies)	⊕⊕⊝⊝ low^a^ ^,^ ^b^	
	121 per 1,000	182 per 1,000 (95–345)				
	Moderate					
	50 per 1,000	76 per 1,000 (40–143)				
DAS28 - IGU only		The mean DAS28-IGU only in the intervention groups was 0.3 lower (0.94 lower to 0.33 higher)		432 (6 studies)	⊕⊕⊝⊝ low^a^ ^,^ ^b^	
AEs - IGU only	Study population		RR 1.18 (0.89–1.56)	1887 (14 studies)	⊕⊕⊝⊝ low^a^ ^,^ ^b^	
	269 per 1,000	317 per 1,000 (239–419)				
	Moderate					
	171 per 1,000	202 per 1,000 (152–267)				

^a^ Downgraded one level due to serious risk of bias (random sequence generation, allocation concealment, blinding, incomplete outcomes) and most of the data comes from the RCTs, with moderate risk of bias.

^b^ Downgraded one level due to the probably substantial heterogeneity.

### 3.5 IGU for AS

#### 3.5.1 BASDAI

**FIGURE 12:**
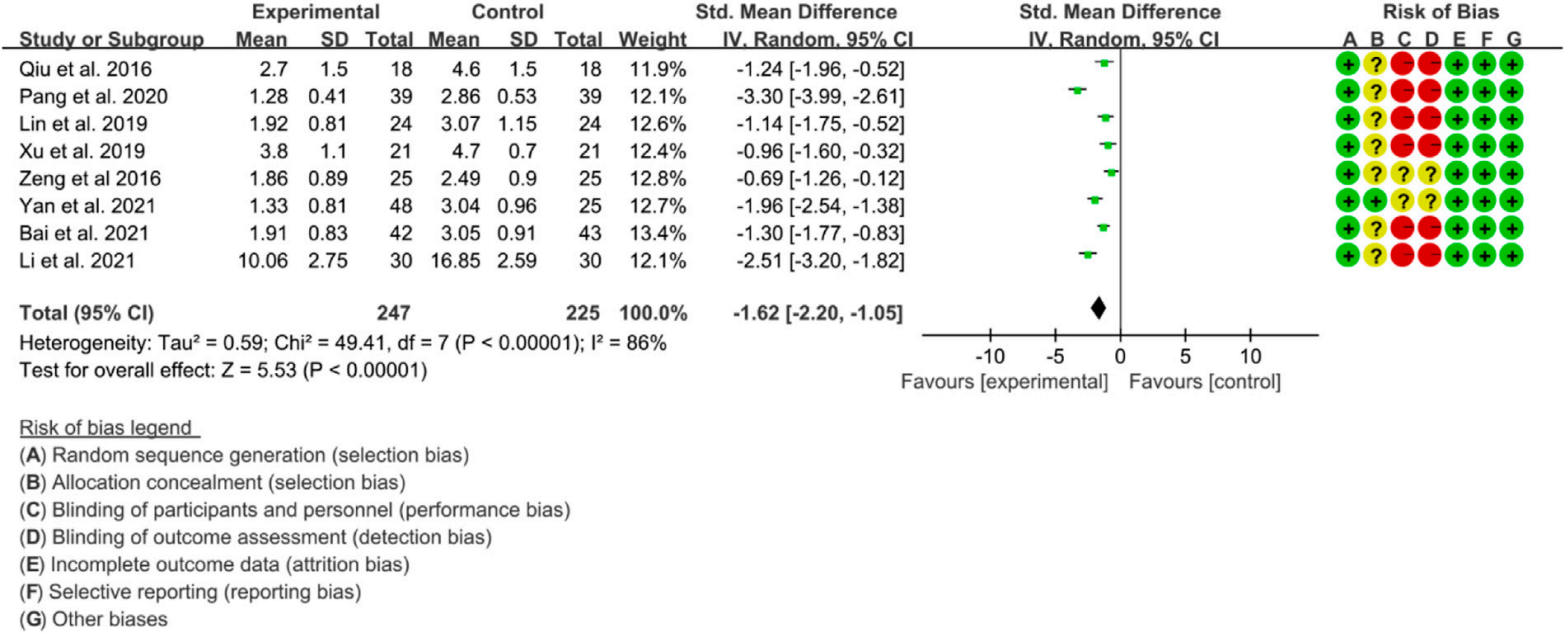
The results of BASDAI.

Eight RCTs used BASDAI as an assessment tool to evaluate the effectiveness of IGU in improving AS. The included studies showed high heterogeneity, with *p* < 0.00001 and I2 = 86%, and thus a random effects model was used for analysis. The meta-analysis results showed that the IGU group had a significantly lower BASDAI score compared to the control group (SMD −1.62 [−2.20, −1.05], *p* < 0.00001; random effect model) (Figure 12). The results of publication bias test showed that it was less likely to have publication bias (*p* = 0.302).

#### 3.5.2 BASFI

**FIGURE 13:**
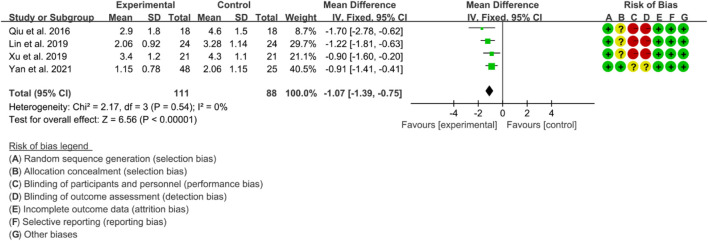
The results of BASFI.

Four RCTs were included in the meta-analysis, all of whom were assessed using BASFI to evaluate the improvement of AS. The heterogeneity test showed low heterogeneity, with *p* = 0.54 and I2 = 0%, indicating that a fixed effects model was appropriate for analysis. The results of the meta-analysis indicated that the IGU group had a significantly lower BASFI score compared to the control group (WMD −1.07 [−1.39, −0.75], *p* < 0.00001; fixed effect model) (Figure 13). The results of publication bias test showed that it was less likely to have publication bias (*p* = 0.254).

#### 3.5.3 VAS

**FIGURE 14:**
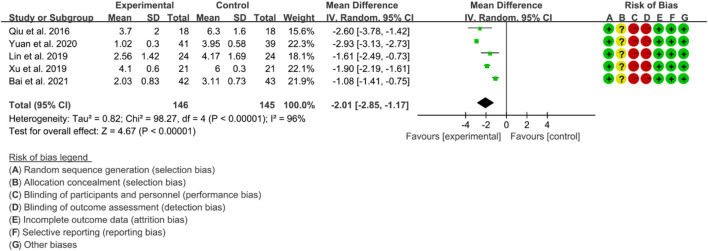
The results of VAS.

Four RCTs were used to evaluate the effect of IGU on the improvement of AS through VAS, with a total of 137 patients in the IGU group and 135 patients in the control group. The heterogeneity test showed significant heterogeneity with *p* < 0.00001 and I2 = 95%, indicating the use of a random effects model for analysis. The meta-analysis results indicated a significant reduction in the VAS score for the IGU group compared to the control group (WMD −2.01 [−2.83, −1.19], *p* < 0.00001; random effects model) (Figure 14). The results of publication bias test showed that it may be likely to have publication bias (*p* = 0.071).

#### 3.5.4 Inflammatory factor

##### 3.5.4.1 Inflammatory factors include ESR, CRP and TNF-α.

**FIGURE 15:**
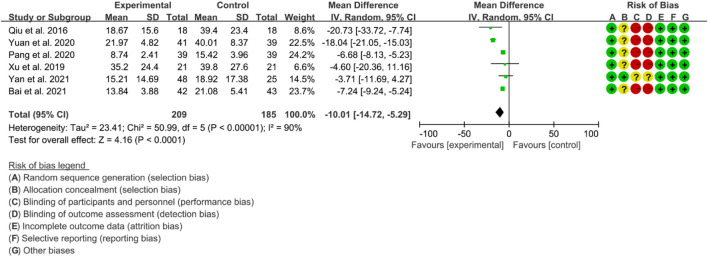
The results of ESR.

Six RCTs were included in the meta-analysis to evaluate the improvement of AS using ESR. High heterogeneity was observed (*p* < 0.00001, I2 = 90%), and therefore, a random effects model was used for the analysis. The results of the meta-analysis showed that the IGU group had a significantly lower ESR compared to the control group (WMD −10.01 [−14.72, −5.29], *p* < 0.0001; random effect model) (Figure 15).

**FIGURE 16:**
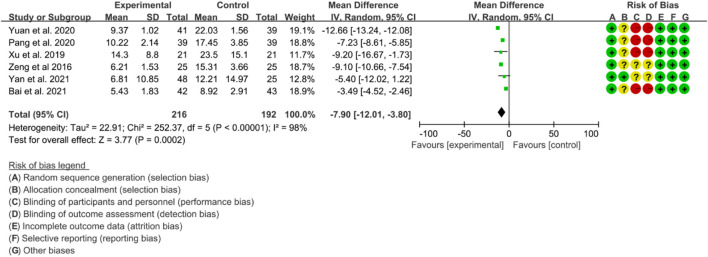
The results of CRP.

Six RCTs were included in the analysis of CRP to evaluate the improvement of AS. The heterogeneity test indicated high heterogeneity (*p* < 0.00001, I2 = 98%), thus a random effects model was utilized for the analysis. The results of the meta-analysis demonstrated that IGU significantly decreased CRP levels compared to the control group (WMD −7.90 [−12.01, −3.80], *p* < 0.00001; random effect model) (Figure 16).

**FIGURE 17:**
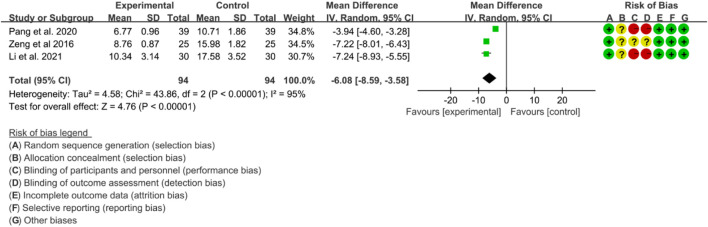
The results of TNF-α

Three RCTs evaluated the effects of IGU on TNF-α levels in the treatment of AS. Significant heterogeneity was detected by the heterogeneity test (*p* < 0.00001, I2 = 95%), and a random effects model was applied for analysis. The results of the meta-analysis indicated that TNF-α levels were significantly lower in the IGU group compared to the control group (WMD -6.08 [-8.59, −3.58], *p* < 0.00001; random effects model) (Figure 17).

#### 3.5.5 Adverse events

**FIGURE 18:**
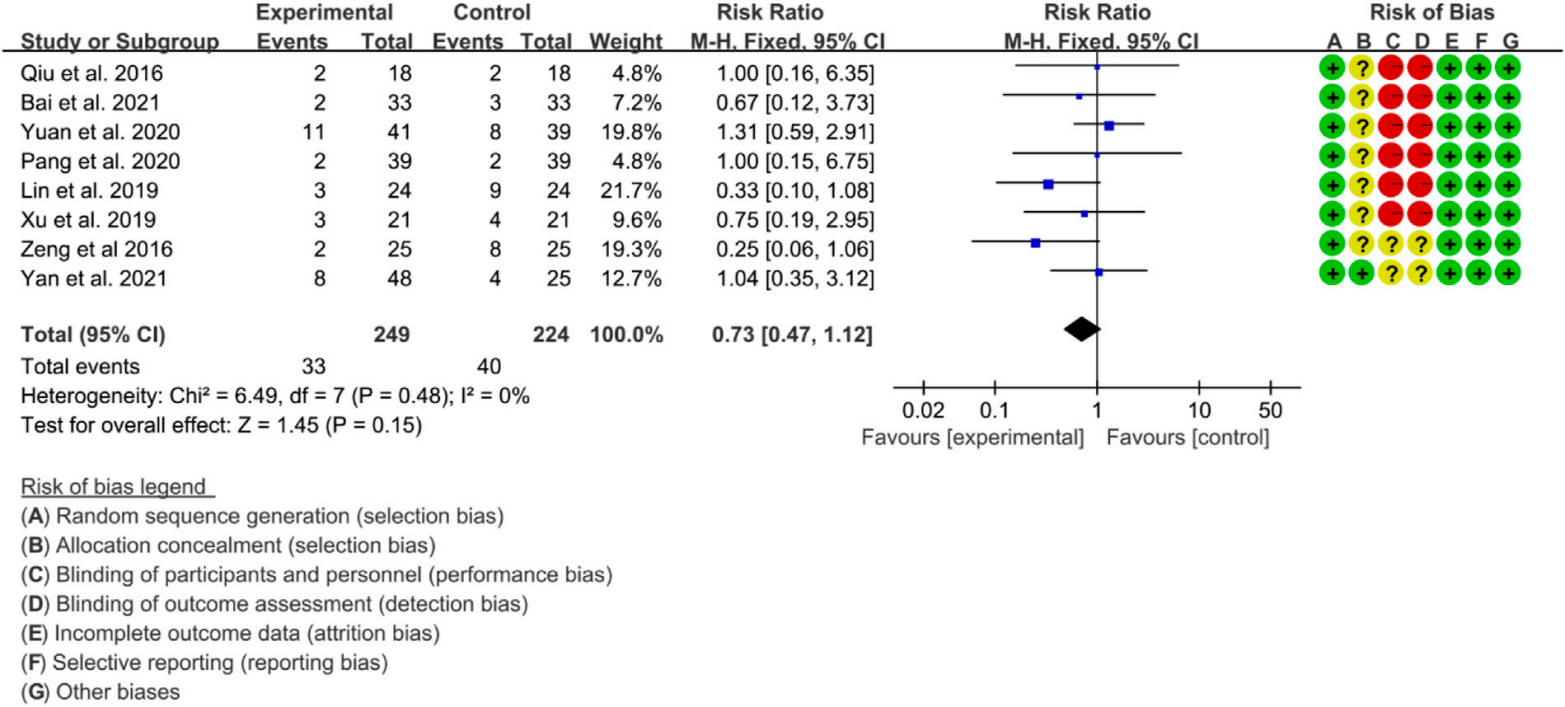
Adverse events.

A total of eight RCTs provided data on adverse events. The heterogeneity test indicated low heterogeneity with *p* = 0.48 and I2 = 0%, suggesting that a fixed effects model was appropriate for analysis. The meta-analysis indicated that there was no significant difference in adverse events between the IGU and control groups (RR 0.72 [0.47, 1.12], *p* = 0.15; fixed effect model) (Figure 18). The results of publication bias test showed that it was less likely to have publication bias (*p* = 0.766).

#### 3.5.6 Quality of evidence

According to the GRADE handbook, the evidence was judged to be moderate to very low (Table 4).

**TABLE 4:** Evidence quality of IGU for AS.

Outcomes	Illustrative comparative risks* (95% CI)		Relative effect (95% CI)	No of participants (studies)	Quality of the evidence (GRADE)	Comments
	Assumed risk	Corresponding risk				
	Control	Adverse event				
BASDAI		The mean basdai in the intervention groups was 1.62 standard deviations lower (2.2–1.05 lower)		472 (8 studies)	⊕⊕⊝⊝ low^a^ ^,^ ^b^	SMD -1.62 (−2.2 to −1.05)
BASFI		The mean basfi in the intervention groups was 1.07 lower (1.39–0.75 lower)		199 (4 studies)	⊕⊕⊕⊝ moderate^a^	
VAS		The mean vas in the intervention groups was 2.01 lower (2.85–1.17 lower)		291 (5 studies)	⊕⊝⊝⊝ very low^a^ ^,^ ^b^ ^,^ ^c^	
Adverse events	Study population		RR 0.73 (0.47–1.12)	473 (8 studies)	⊕⊕⊕⊝ moderate^a^	
	179 per 1,000	130 per 1,000 (84–200)				
	Moderate					
	175 per 1,000	128 per 1,000 (82–196)				

^a^ Downgraded one level due to serious risk of bias (random sequence generation, allocation concealment, blinding, incomplete outcomes) and most of the data comes from the RCTs, with moderate risk of bias.

^b^ Downgraded one level due to the probably substantial heterogeneity.

^c^ Downgraded one level due to potential publication bias.

### 3.6 IGU for PSS

#### 3.6.1 ESSPRI

**FIGURE 19:**
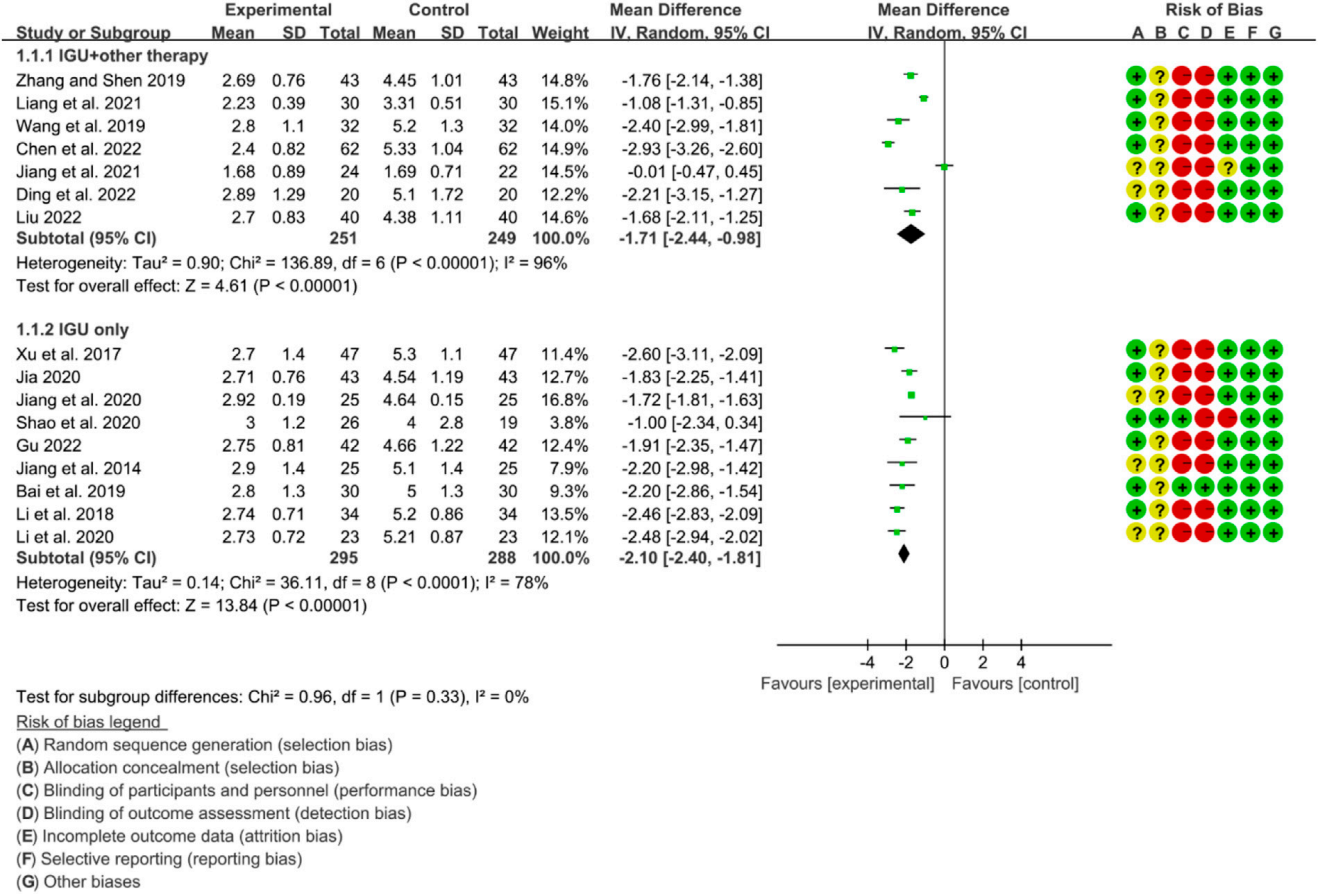
Esspri.

The heterogeneity test showed that some subgroups had high heterogeneity (IGU + other therapy subgroup: *p* < 0.00001, I2 = 96%; IGU only subgroup: *p* < 0.0001, I2 = 78%), and a random effect model was used. The meta-analysis results show that compared with the control group, the ESSPRI in the IGU + other therapy group (WMD −1.71 [−2.44, −0.98], *p* < 0.00001; random effect model) and IGU only group (WMD −2.10 [−2.40, −1.81], *p* < 0.00001; random effect model) was lower (Figure 19). The results of publication bias test showed that it was less likely to have publication bias in IGU + other therapy subgroup (*p* = 0.667), while the publication bias test showed that it was likely to have publication bias in IGU only subgroup (*p* = 0.066).

#### 3.6.2 ESSDAI

**FIGURE 20:**
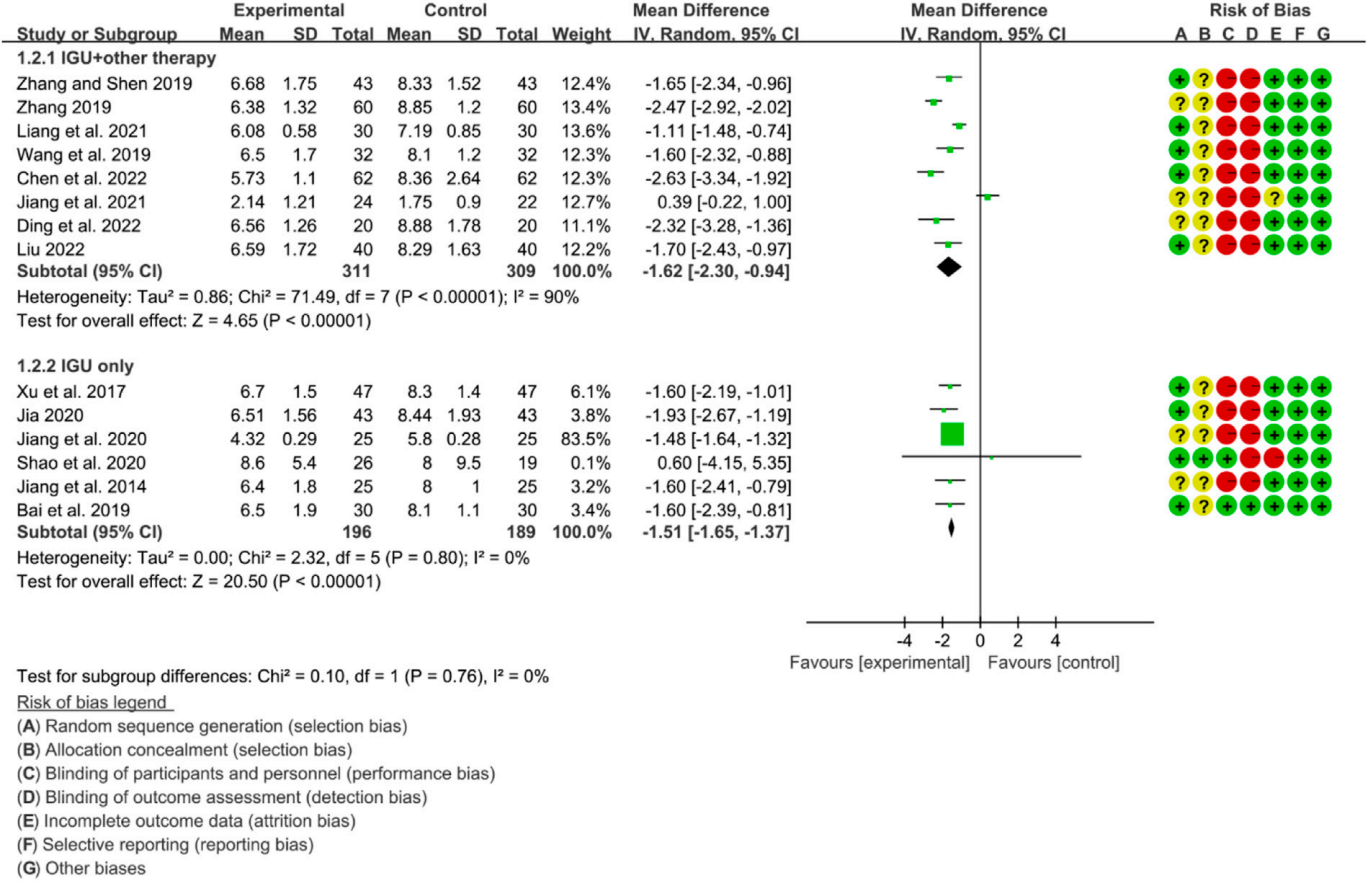
Essdai.

The heterogeneity test showed that some subgroups had high heterogeneity (IGU + other therapy subgroup: *p* < 0.00001, I2 = 90%; IGU only subgroup: *p* = 0.80, I2 = 0%), and a random effect model was used. The meta-analysis results show that compared with the control group, the ESSDAI in the IGU + other therapy group (WMD −1.62 [−2.30, −0.94], *p* < 0.00001; random effect model) and IGU only group (WMD −1.51 [−1.65, −1.37], *p* < 0.00001; random effect model) was lower (Figure 20). The results of publication bias test showed that it was less likely to have publication bias in IGU + other therapy (*p* = 0.691) and IGU only subgroup (*p* = 0.659).

#### 3.6.3 Schirmer’s test

**FIGURE 21:**
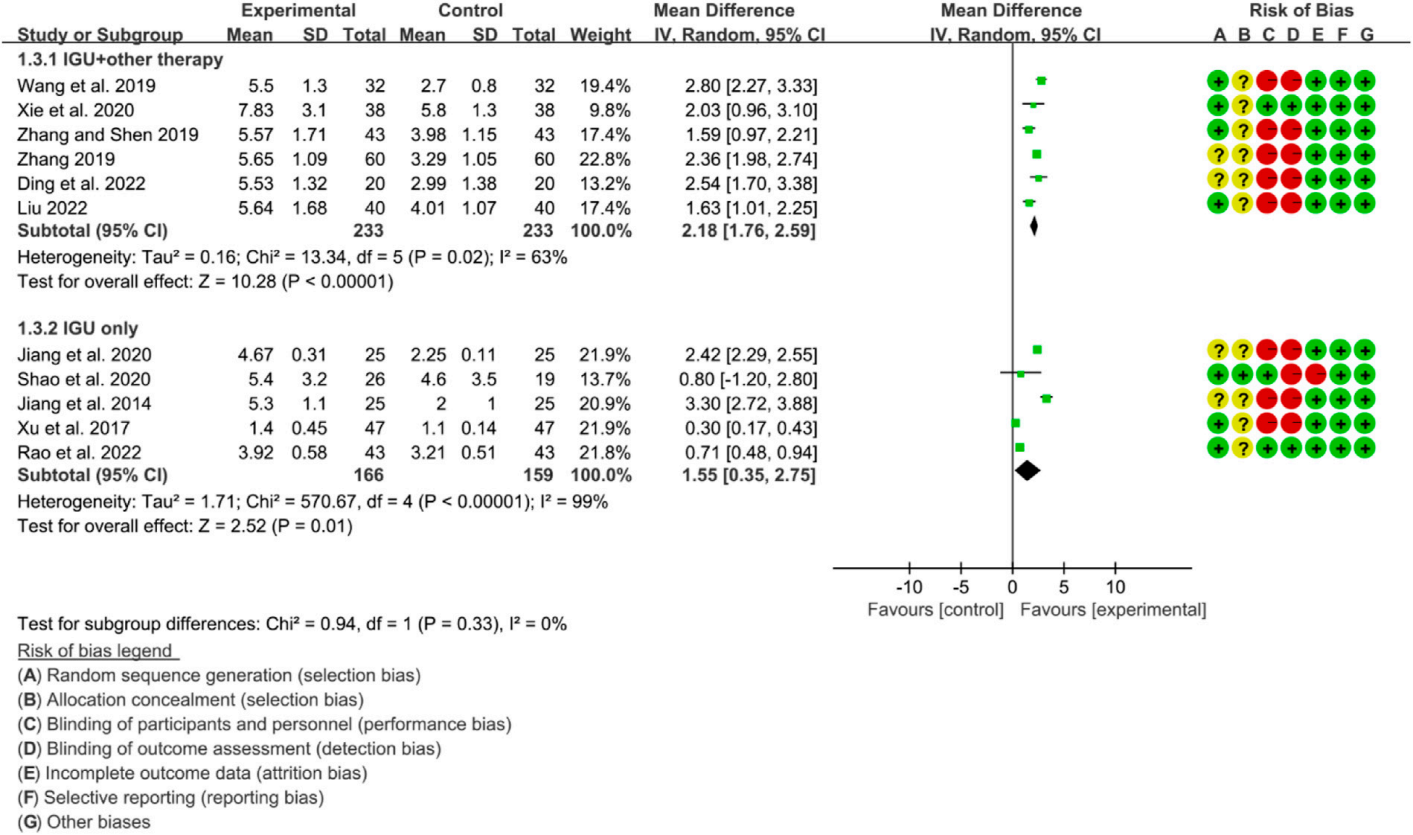
Schirmer’s test.

The heterogeneity test showed that some subgroups had high heterogeneity (IGU + other therapy subgroup: *p* = 0.02, I2 = 63%; IGU only subgroup: *p* < 0.00001, I2 = 99%), and a random effect model was used. The meta-analysis results show that compared with the control group, the schirmer’s test in the IGU + other therapy group (WMD 2.18 [1.76, 2.59], *p* < 0.00001; random effect model) and IGU only group (WMD 1.55 [0.35, 2.75], *p* = 0.01; random effect model) was higher (Figure 21). The results of publication bias test showed that it was less likely to have publication bias in IGU + other therapy (*p* = 0.612) and IGU only subgroup (*p* = 0.934).

#### 3.6.4 Inflammation factors

Inflammation factors include ESR, CRP and RF.

**FIGURE 22:**
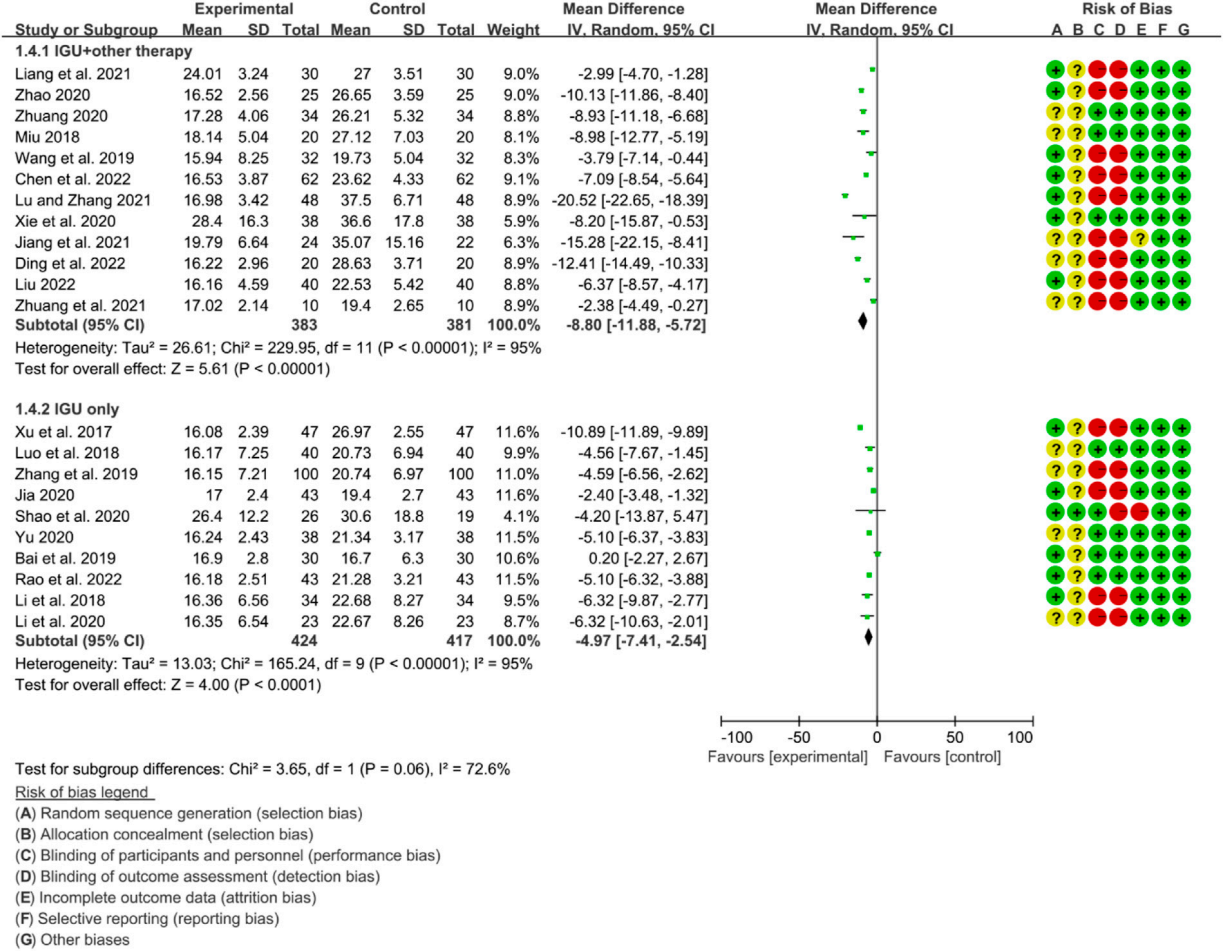
Esr.

For ESR, the heterogeneity test showed that some subgroups had high heterogeneity (IGU + other therapy subgroup: *p* < 0.00001, I2 = 95%; IGU only subgroup: *p* < 0.00001, I2 = 95%), and a random effect model was used. The meta-analysis results show that compared with the control group, the ESR in the IGU + other therapy group (WMD −8.80 [−11.88, −5.72], *p* < 0.00001; random effect model) and IGU only group (WMD −4.97 [−7.41, −2.54], *p* < 0.0001; random effect model) was lower (Figure 22).

**FIGURE 23:**
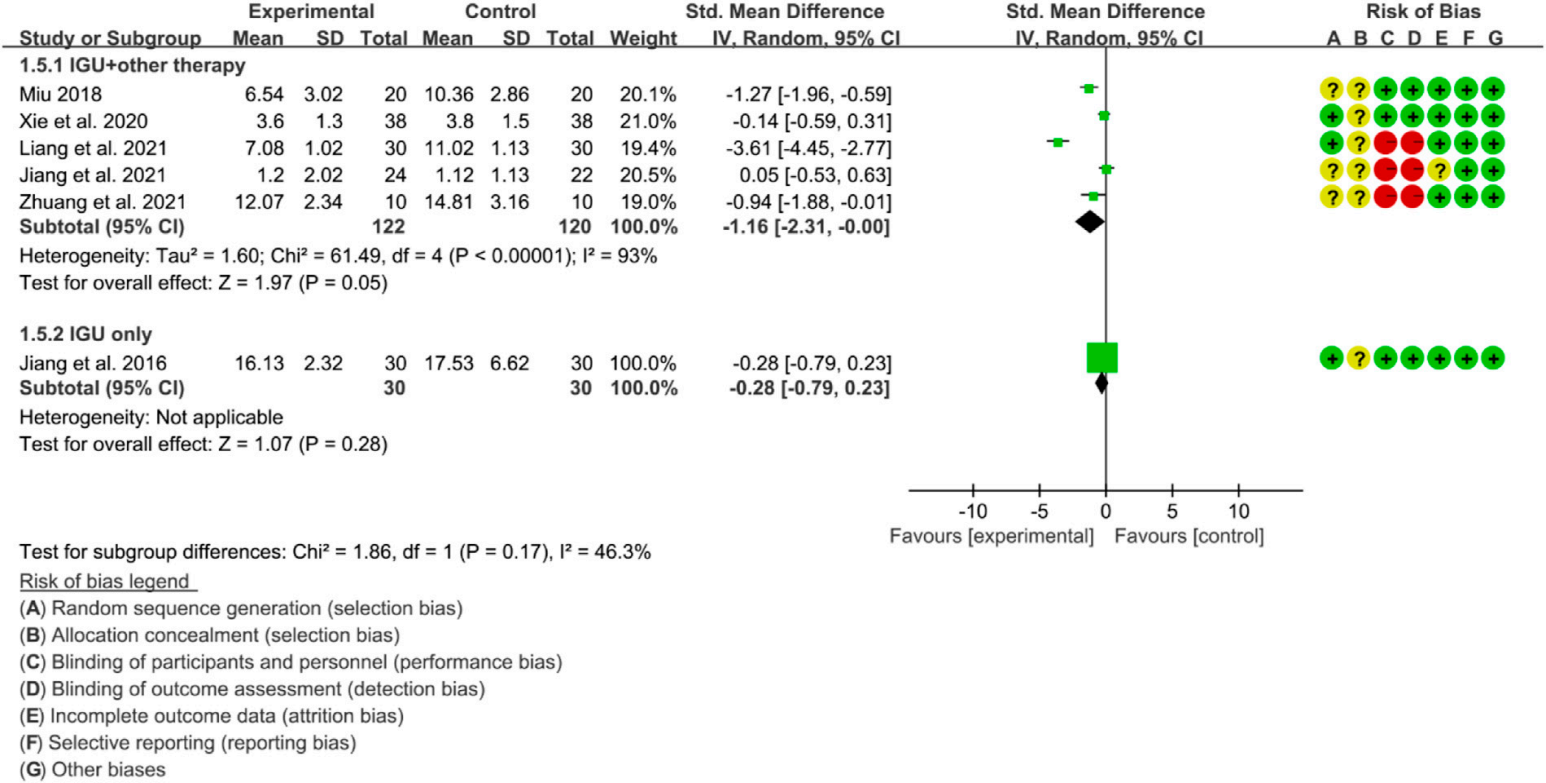
Crp.

For CRP, the heterogeneity test showed that some subgroups had high heterogeneity (IGU + other therapy subgroup: *p* < 0.00001, I2 = 93%; IGU only subgroup: not applicable), and a random effect model was used. The meta-analysis results show that compared with the control group, the CRP in the IGU + other therapy group was lower (SMD −1.16 [−2.31, −0.00], *p* = 0.05; random effect model) (Figure 23).

**FIGURE 24:**
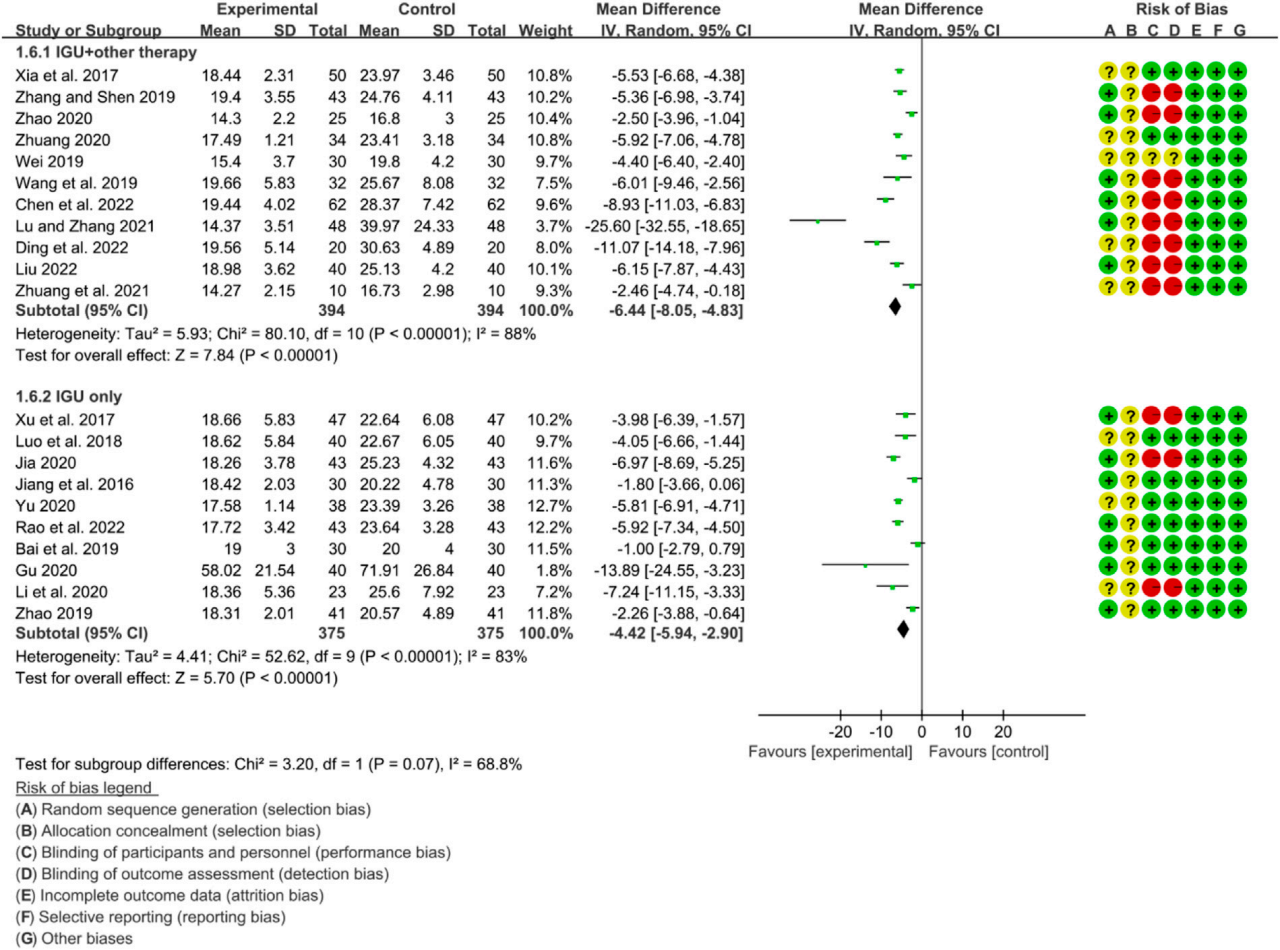
Rf.

For RF, the heterogeneity test showed that some subgroups had high heterogeneity (IGU + other therapy subgroup: *p* < 0.00001, I2 = 88%; IGU only subgroup: *p* < 0.00001, I2 = 83%), and a random effect model was used. The meta-analysis results show that compared with the control group, the RF in the IGU + other therapy group (WMD −6.44 [−8.05, −4.83], *p* < 0.00001; random effect model) and IGU only group (WMD −4.42 [−5.94, −2.90], *p* < 0.0001; random effect model) was lower (Figure 24).

#### 3.6.5 Adverse events

**FIGURE 25:**
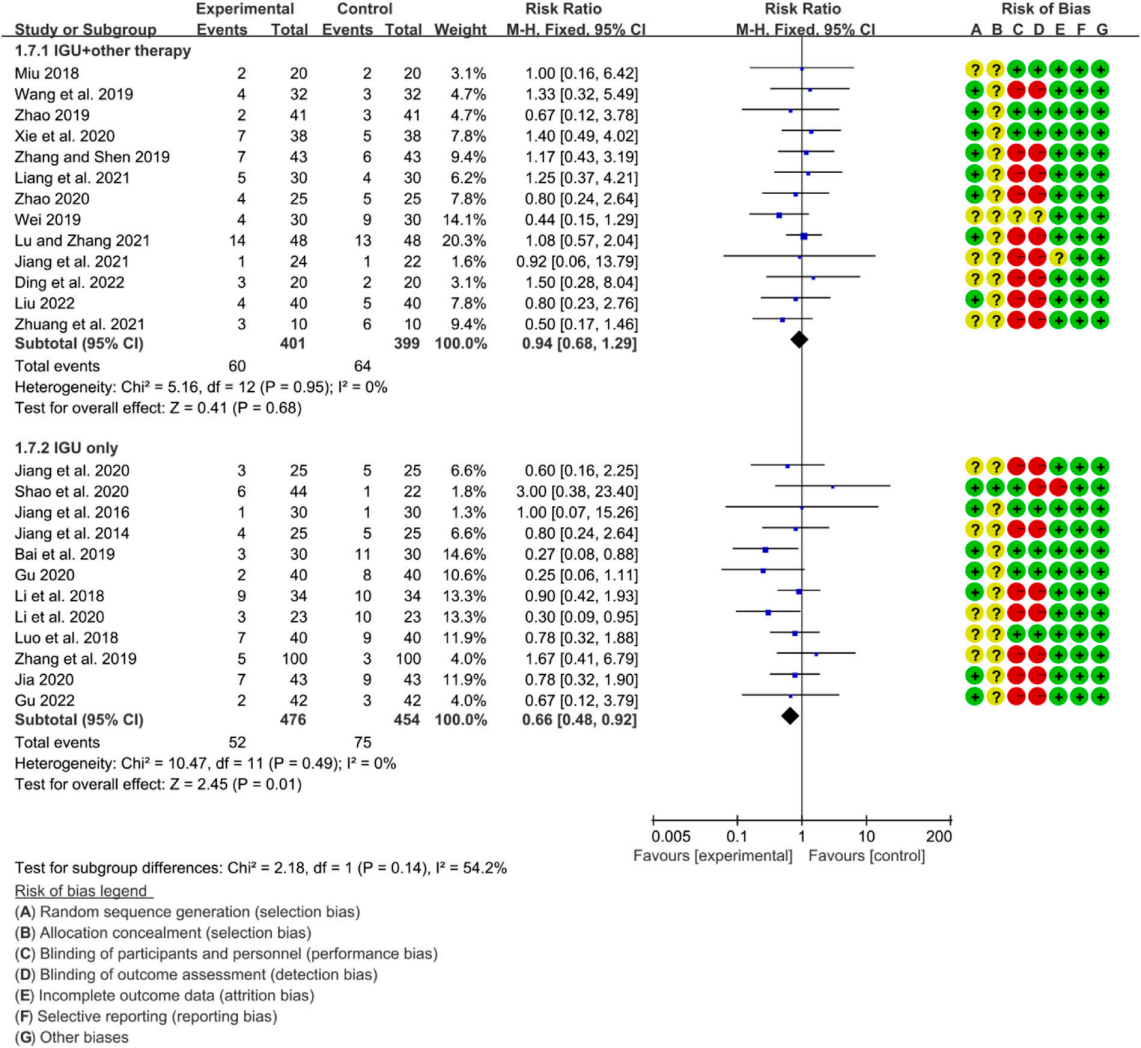
Adverse events.

The heterogeneity test showed that some subgroups had high heterogeneity (IGU + other therapy subgroup: *p* = 0.95, I2 = 0%; IGU only subgroup: *p* = 0.49, I2 = 0%), and a fixed effect model was used. The meta-analysis results show that compared with the control group, the incidence of adverse events in the IGU only group (RR 0.66 [0.48, 0.98], *p* = 0.01; fixed effect model) was lower, while the difference of the incidence of adverse events between IGU + other therapy group and control grouo was of no statistical significance (RR 0.94 [0.68, 1.29], *p* = 0.68; fixed effect model) was lower (Figure 25). The results of publication bias test showed that it was less likely to have publication bias in IGU + other therapy (*p* = 0.777) and IGU only subgroup (*p* = 0.501).

#### 3.6.6 Quality of evidence

According to the GRADE handbook, the evidence of IGU + other therapy subgroup was judged to be moderate to low (Table 5). The evidence of IGU only subgroup was judged to be moderate to very low (Table 6).

**TABLE 5:** Evidence quality of IGU for PSS in IGU + other therapy subgroup.

Outcomes	Illustrative comparative risks* (95% CI)		Relative effect (95% CI)	No of participants (studies)	Quality of the evidence (GRADE)	Comments
	Assumed risk	Corresponding risk				
	Control	Primary outcomes				
ESSPRI - IGU + other therapy		The mean ESSPRI in the intervention groups was 1.71 lower (2.44–0.98 lower)		500 (7 studies)	⊕⊕⊝⊝ low^a^ ^,^ ^b^	
ESSDAI - IGU + other therapy		The mean ESSDAI in the intervention groups was 1.62 lower (2.3–0.94 lower)		620 (8 studies)	⊕⊕⊝⊝ low^a^ ^,^ ^b^	
Schirmer’s test - IGU + other therapy		The mean Schirmer’s test in the intervention groups was 2.18 higher (1.76–2.59 higher)		466 (6 studies)	⊕⊕⊝⊝ low^a^ ^,^ ^b^	
Advers events - IGU + other therapy	Study population		RR 0.94 (0.68–1.29)	800 (13 studies)	⊕⊕⊕⊝ moderate^a^	
	160 per 1,000	151 per 1,000 (109–207)				
	Moderate					
	132 per 1,000	124 per 1,000 (90–170)				

^a^ Downgraded one level due to serious risk of bias (random sequence generation, allocation concealment, blinding, incomplete outcomes) and most of the data comes from the RCTs, with moderate risk of bias.

^b^ Downgraded one level due to the probably substantial heterogeneity.

**TABLE 6:** Evidence quality of IGU for PSS in IGU only subgroup.

Outcomes	Illustrative comparative risks* (95% CI)		Relative effect (95% CI)	No of participants (studies)	Quality of the evidence (GRADE)	Comments
	Assumed risk	Corresponding risk				
	Control	Adverse event				
ESSPRI - IGU only		The mean ESSPRI in the intervention groups was 2.1 lower (2.4–1.81 lower)		583 (9 studies)	⊕⊝⊝⊝ very low^a^ ^,^ ^b^ ^,^ ^c^	
ESSDAI - IGU only		The mean ESSDAI in the intervention groups was 1.51 lower (1.65–1.37 lower)		385 (6 studies)	⊕⊕⊕⊝ moderate^a^	
Schirmer’s test - IGU only		The mean schirmer’s test in the intervention groups was 1.55 higher (0.35–2.75 higher)		325 (5 studies)	⊕⊕⊝⊝ low^a^ ^,^ ^b^	
Adverse events - IGU only	Study population		RR 0.66 (0.48–0.92)	930 (12 studies)	⊕⊕⊕⊝ moderate^a^	
	165 per 1,000	109 per 1,000 (79–152)				
	Moderate					
	200 per 1,000	132 per 1,000 (96–184)				

^a^ Downgraded one level due to serious risk of bias (random sequence generation, allocation concealment, blinding, incomplete outcomes) and most of the data comes from the RCTs, with moderate risk of bias.

^b^ Downgraded one level due to the probably substantial heterogeneity.

^c^ Downgraded one level due to potential publication bias.

### 3.7 IGU for autoimmune disease with interstitial pneumonia

Zhuang et al. (2021) and Zhang et al. (2019) reported the treatment of PSS with interstitial pneumonia. DongZhang et al. (2019) reported the treatment of RA with interstitial pneumonia. Zhang et al. (2019) and DongZhang et al. (2019) reported FVC; they found that IGU may improve FVC.

Meanwhile, Zhuang et al. (2021) showed that both DLCO and 6MWT improved in both groups after treatment, and the degree of improvement in 6MWT in the IGU group was due to that in the control group. Zhang et al. (2019) reported that MMF was also improved after treatment, and the improvement was greater in the IGU group than in the control group. DongZhang et al. (2019) showed that compared with the control group, both FEV1 and TLC were improved after IGU treatment (*p* < 0.05).

## 4 Discussion

### 4.1 IGU for RA

IGU was approved for the treatment of RA in China and Japan in 2012, and in the RA guidelines of the Asia Pacific Association of Rheumatology (APLAR) meeting in 2014. It is recommended as an effective option for intensive treatment of refractory RA (Li et al., 2013; Li J. et al., 2019). It is now widely used to treat autoimmune diseases and improve related inflammation, such as PSS, IgG4-related diseases, lupus nephritis, *etc.* (Nozaki, 2021). Studies have shown that compared with other traditional DMARDs drugs, IGU can not only inhibit the production of immunoglobulin and various inflammatory cytokines (IL-1, IL-6, IL-8 and TNF), promote the differentiation of bone cells, inhibit the generation of osteoclasts, reduce bone resorption and joint destruction, but also reduce the expression of matrix metalloproteinases by inhibiting the production of MMP-1 and MMP-3, thereby playing an anti-inflammatory role (Liu et al., 2021a; Mizutani et al., 2021; Mu et al., 2021; Tanaka, 2021). In addition, IGU can also inhibit COX-2 and reduce the short-term synergistic effect of pain and inflammation (Mu et al., 2021; Tanaka, 2021).

This meta-analysis found that IGU + MTX therapy can improve ACR20, ACR50, ACR70, DAS28, reduce ESR, CRP, RF, and have a lower incidence of adverse events than the control group. However, IGU alone only significantly improved CRP. IGU + Tripterygium Extract can also improve ESR, CRP and RF. This suggests that IGU + MTX may be a better combination of IGU in the treatment of RA, because it has obvious efficacy, can reduce inflammatory factors, and has a lower incidence of adverse events than the control group therapy (mainly MTX). There is heterogeneity in most outcomes, which is considered to be related to the following points: 1) the dose and duration of IGU and MTX are different; 2) the degree of disease activity of patients at baseline is not the same. Since the extent of disease activity in patients at baseline was not clearly stated in each study, further analysis was not performed. In addition, the dose of IGU in all RCTs was 25–50 mg (25 mg Bid for most RCTs; and 25 Qd or 50 mg Qd for a few RCTs), suggesting that IGU at this dose had a good effect on RA without increasing the incidence of adverse events.

A recent 52-week randomized, double-blind, parallel-controlled, multicenter study by Bao et al. showed that IGU (Use alone) was more effective than MTX in the treatment of RA (Du F. et al., 2021). In terms of efficacy, the ACR20 response rate of IGU was 77.44%, which was significantly better than that of MTX (65.87%). In the direction of imaging improvement, the results showed that the proportion of patients with no imaging progression in IGU or combined therapy for 1 year was higher than that in MTX therapy, indicating that IGU therapy was significantly better than MTX therapy. The efficacy of IGU + MTX is similar to that of IGU only, suggesting that patients with early RA can consider IGU alone, and only when the single drug is not effective, combined with other drugs such as biological agents. They also found that IGU or combination therapy can delay the imaging progress of RA patients, which provides an important reference for clinical medication. Another important factor for RA patients and doctors when choosing a drug is the efficacy, safety and cost of the drug. Jie et al. reported data from a real-world pharmacoeconomics study on IGU and other drugs in RA at the 2022 EULAR meeting. Their results show that IGU combined with MTX in the treatment of RA is both safe and effective, and the price is moderate, providing a treatment plan for RA patients that takes into account efficacy, safety and economic cost.

### 4.2 IGU for AS

The current study shows that IGU, as a new type of DMARD, mainly acts through anti-inflammatory and immune regulation. For example, IGU can inhibit the production of inflammatory cytokines (such as IL-1 and TNF-α), block the IL-17 signaling pathway and inhibit cyclooxygenase, and regulate the balance of osteoclasts (Liu et al., 2021b; Harjacek, 2021), so it may be effective against AS/SpA in mechanism. Therefore, a number of exploratory RCTs have previously applied IGU to AS/SpA (Qiu et al., 2016; Zeng et al., 2016; Lin et al., 2019; Xu et al., 2019; Pang et al., 2020; Yuan et al., 2020; Li Y. et al., 2021; Bai et al., 2021; Li X. et al., 2021).

The meta-analysis findings revealed that IGU was effective in reducing the BASDAI score, BASFI score, and VAS. Additionally, IGU was able to lower inflammation levels by decreasing ESR, CRP, and TNF-α. However, there was considerable heterogeneity in the results, especially in VAS, ESR, CRP, and TNF-α. This could be attributed to the fact that BASDAI and VAS are subjective measures, and the experiences of patients across different RCTs may differ. Moreover, ESR, CRP, and TNF-α are individual biochemical indicators, and variations in patients’ conditions across different RCTs may also contribute to the heterogeneity. All RCTs reported adverse events, but no patient deaths were recorded. Compared to the control group, the IGU group did not experience any statistically significant difference in adverse events. Therefore, IGU does not appear to increase the risk of adverse events. Notably, the IGU dose was 50 mg in all RCTs (25 mg Bid in most RCTs and 50 mg Qd in a few RCTs), indicating that this dose had a beneficial effect on AS without raising the incidence of adverse events.

### 4.3 IGU for PSS

The pathogenesis of PSS is complex and has not yet been clearly studied. At present, it is believed that it may be related to various factors such as genetics, environment, endocrine, and immune abnormalities (Fasano et al., 2020a; Huang et al., 2021). Among them, the excessive activation of B cells produces a variety of autoantibodies and hyperimmunoglobulinemia plays an important role in the development of pSS. In this process, T cells also participate in the maturation and differentiation of B cells by secreting a variety of cytokines (Rivière et al., 2020). More than 80% of patients with Sjögren’s syndrome will experience symptoms of dryness, fatigue and joint pain, which will affect the patient’s work efficiency and reduce the patient’s quality of life (Marshall and Stevens, 2018). However, there is currently no specific drug for the treatment of pSS. Therefore, exploratory research on PSS therapeutic drugs is currently underway (Carsons et al., 2017; Vehof et al., 2020). As a new type of DMARD, IGU’s main mechanism of action is highly compatible with the complex pathogenesis of SS, and has therapeutic potential. A number of clinical studies have shown that IGU can effectively improve the disease activity (such as ESSDAI), various serum indicators (IgG, IgM, IgA, ESR, RF) and lacrimal gland secretion function (detected by Schirmer I test) in patients with pSS.

This meta-analysis also showed that IGU can reduce the ESSPRI score and ESSDAI score, inhibit the inflammation factors (reduce ESR, CRP and RF) and increase Schirmer’s test score. The incidence of adverse events in IGU group was also lower than that in control group, indicating that the addition of IGU may be an effective and safe treatment plan. In addition, the dose of IGU in all RCTs was 50 mg (25 mg Bid for most RCTs and 50 mg Qd for a few RCTs), suggesting that IGU at this dose had a good effect on PSS without increasing the incidence of adverse events. B cell hyperactivity is a key pathogenic factor in pSS, which is mainly characterized by the formation of ectopic germinal centers in the lacrimal and salivary glands (Carsons et al., 2017; Fasano et al., 2020b; Du W. et al., 2021). Therefore, reducing B cell activity and suppressing immunoglobulin production have become the key to treatment. Studies have shown that IGU not only inhibits the proliferation of T cells, but also inhibits the differentiation of antibody secreting cells (ASCs) in RA patients by activating the PKC/EGR1 pathway, thereby regulating the immune response of B cell differentiation and relieving clinical symptoms (Ye et al., 2019a). However, whether IGU can play a role in the treatment of pSS patients by inhibiting the activity of B cells has not yet been determined.

### 4.4 IGU for interstitial pneumonia

Early symptoms of RA-interstitial pneumonia (RA-ILD) are often atypical and easy to miss (Chernau et al., 2019; Graney and Fischer, 2019). At present, there is no targeted treatment for RA-ILD, and two clinical strategies are mainly used: anti-inflammatory and anti-fibrosis. In terms of anti-inflammatory, the dosage and treatment time of hormones and immunosuppressants are difficult to grasp. Excessive immunosuppression can also lead to secondary infection aggravating the disease. Therefore, clinical studies are still searching for safe and effective therapeutic drugs for RA-ILD (Wells and Denton, 2014; Santhanam et al., 2020). The current study shows that the potential mechanisms of IGU treatment of pulmonary fibrosis include: inhibition of inflammation and epithelial-mesenchymal transition (EMT) process (Luppi et al., 2020). For example, Luo et al. found that inflammatory cell infiltration, inflammatory factor and chemokine expression in the lung tissue of mice treated with IGU treated mice with idiopathic pulmonary fibrosis decreased in a dose-dependent manner. This suggests that IGU can inhibit the pulmonary inflammatory response that accompanies the process of pulmonary fibrosis (Yoo et al., 2020). Zhao et al. found that high doses of IGU and methylprednisolone had inhibitory effects on alveolitis and pulmonary fibrosis in a bleomycin-induced mouse model of pulmonary fibrosis (England and Hershberger, 2020). Zhu et al. found that IGU can inhibit TGF-β1-mediated human lung fibroblast activation and collagen secretion through the Smad3/p300 pathway, and it may be an effective anti-fibrotic drug to delay the progression of PF (Kadura and Raghu, 2021).

In this systematic review and meta-analysis, Zhuang et al. (2021) and Zhang et al. (2019) reported the treatment of PSS with interstitial pneumonia. DongZhang et al. (2019) reported the treatment of RA with interstitial pneumonia. The meta-analysis results showed that FVC increased after IGU treatment. Meanwhile, Zhuang et al. (2021) showed that both DLCO and 6MWT improved in both groups after treatment, and the degree of improvement in 6MWT in the IGU group was due to that in the control group. Zhang et al. (2019) reported that MMF was also improved after treatment, and the improvement was greater in the IGU group than in the control group. DongZhang et al. (2019) showed that compared with the control group, both FEV1 and TLC were improved after IGU treatment. These all suggest the therapeutic effect of IGU on autoimmune diseases complicated with interstitial pneumonia. In terms of economics and drug insurance policy, IGU is a relatively inexpensive drug that is available in most countries. A real-world study retrospectively analyzed the population characteristics, efficacy and influencing factors of RA patients who received IGU treatment for at least 6 months between July 2015 and October 2020 and had more than 3 follow-up records. The results showed that IGU was well tolerated and an effective treatment drug, which is a treatment option for RA patients with interstitial lung disease.

### 4.5 IGU for other rheumatic and autoimmune diseases

SLE is an autoimmune inflammatory disease that affects multiple organs and connective tissues. It is more common in young women and is seeing an increase in early, mild, and atypical cases (Luo et al., 2015; Shao et al., 2021). Within 5 years, most SLE patients will develop LN, which remains a significant cause of morbidity and mortality (Zhao et al., 2017b). While several drugs have demonstrated efficacy in treating the disease, 20%–35% of LN patients experience relapse or treatment failure, and drug intolerance is a frequent issue (Fu et al., 2021). In preclinical studies with lupus, IGU prevented autoimmune nephritis, reduced proteinuria, and decreased immune complex deposition in MRL/lpr mice (Anders et al., 2020). As the most critical pathogenic cells in the progression and development of systemic lupus erythematosus, B cells are closely related to the systemic damage and antibody secretion of SLE (Gasparotto et al., 2020; Ayoub and Nachman, 2021). The earliest study on the mechanism of IGU on B cell differentiation found that it can inhibit the production of immunoglobulin by B cells (Mahajan et al., 2020). In a phase III clinical trial in RA, IGU reduced serum immunoglobulin concentrations (Yan et al., 2014; Canny and Jackson, 2021). In animal models of RA and lupus, IGU reduced autoantibody titers, including anti-collagen antibodies (Tanaka et al., 2003; Ma et al., 2019) and anti-double-stranded (dsDNA) antibodies [198]. Interestingly, IGU has been reported to reduce peripheral plasma cell counts without affecting the total B cell population in MRL/lpr mice (Anders et al., 2020). Further studies have shown that in RA patients receiving IGU only, IGU regulates key transcription factors affecting plasma cell differentiation through the PKC/Egr1 axis, especially Blimp-1 (Hara et al., 2007). A recent observational study found that more than 90% of patients with refractory LN responded to IGU within 24 weeks without the need to increase steroid dosage or add any other drugs during follow-up (Lu et al., 2009). Yan et al. are currently conducting a multicenter, randomized, 52-week parallel active drug-controlled study (Du et al., 2008). The study aims to investigate the efficacy of iguratimod as first-line treatment for patients with LN. Patients with biopsy-proven active lupus nephritis from six study sites in China were randomly assigned to the experimental or control group. During the first 24 weeks, IGU was compared to cyclophosphamide as induction therapy, while during the second 24 weeks, IGU was compared to azathioprine as maintenance therapy. The primary outcome was the rate of renal response, including complete and partial response at week 52, which will be analyzed using a noninferiority hypothesis test. This ongoing trial will determine whether iguratimod can be used as an alternative induction or maintenance therapy for lupus nephritis patients (Du et al., 2008).

**FIGURE 26:**
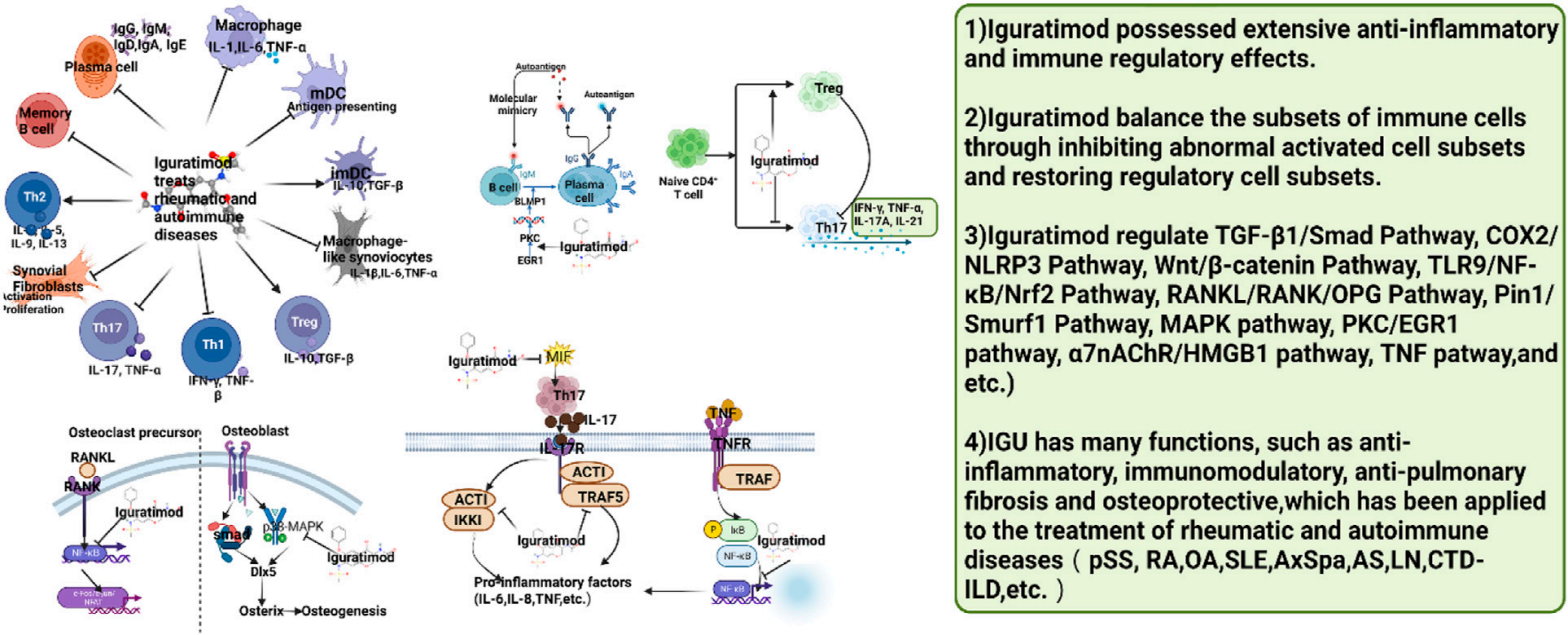
Summary of the mechanism of IGU treatment of rheumatic and autoimmune diseases (IGU may regulate immune cell function and activity to balance immune cell subsets, thereby further reducing inflammation and tissue damage. pSS, primary Sjögren’s syndrome; RA, rheumatoid arthritis; OA, Osteoarthritis; SLE, systemic lupus erythematosus; AS, ankylosing spondylitis; LN, lupus nephritis; CTD-ILD, connective tissue disease associated interstitial lung disease).

In summary, the mechanism of IGU treatment of rheumatic and autoimmune diseases is summarized in Figure 26.

### 4.6 Strengths and limitations

Compared with previous systematic reviews and meta-analyses, the strengths of this study are: 1) Compared with previous studies on PSS (Luo et al., 2013; Pu et al., 2021), this study included newer and more RCTs (32, 5 of which were published in 2022), and the quality of evidence was assessed. 2) Compared with previous studies on RA (Ye et al., 2019b; Kang et al., 2020; Shrestha et al., 2020; Hu et al., 2021; Shrestha et al., 2021; Yan et al., 2021; Zeng et al., 2022a; Zeng et al., 2022b; Long et al., 2023), this study also included newer and more RCTs (43, 4 of which were published in 2022); and the intervention in the IGU group is IGU alone or IGU combined with other drugs, not limited to IGU + MTX, and further found that the combination of IGU + MTX may reduce the occurrence of adverse events, while IGU combined with other drugs only does not increase adverse events. 3) Compared with previous studies on AS (Chen et al., 2021; Liu B. et al., 2021; Deng et al., 2022; Ouyang et al., 2022; Long et al., 2023), this research employed a more rigorous screening process for RCTs. Moreover, this systematic review and meta-analysis integrated findings from various rheumatic and autoimmune diseases. As a result, the efficacy of IGU treatment for AS can be cross-compared with the outcomes of IGU treatment for other rheumatic and autoimmune diseases. 4) This study also evaluated the efficacy and safety of IGU in the treatment of autoimmune disease with interstitial pneumonia for the first time. 5) This study performed a thorough search of different databases and included Chinese databases.

The limitations include: 1) Although there is no language restriction, most of the included RCTs are in Chinese and English, and no literature in other languages has been found, so there may be publication bias. 2) The basic treatment, course of treatment, and observation time of the indicators are also different, and the clinical heterogeneity among the subgroups is high, which leads to a decrease in the accuracy and implementability of the results. 3) Although 84 RCTs were included, only 4 types of diseases (RA, AS, PSS and Autoimmune disease with interstitial pneumonia) were involved, and RCTs of IGU for other rheumatic and autoimmune diseases were not retrieved. 4) Since RCTs did not report on patients’ disease conditions in detail (such as naive RA and MTX-resistant RA), subgroup analysis of patients’ disease conditions could not be performed. 5) The RCTs included in this study are all in English or Chinese, and there are no literature in other languages (such as Japanese) for the time being, which may lead to potential bias. 6) The quality of evidence for most outcomes was assessed as low to very low, which may affect the generalization of conclusions.

Based on these shortcomings, more IGUs are needed in the future for RCTs of other rheumatic and autoimmune diseases (such as SLE). Furthermore, future RCTs are expected to report more detailed patient medication information to facilitate subgroup analysis and reduce clinical heterogeneity.

## 5 Conclusion

Based on current evidence, IGU may be a safe and effective for the treatment of RA, AS, PSS and autoimmune diseases with interstitial pneumonia. The quality of evidence was very low to moderate. The recommended dose is 25–50 mg. However, more RCTs about other type of rheumatic and autoimmune diseases are still needed.

## Data Availability

The original contributions presented in the study are included in the article/Supplementary Material, further inquiries can be directed to the corresponding authors.
